# Revealing the transitory and local effect of zebularine on development and on proteome dynamics of *Salix purpurea*

**DOI:** 10.3389/fpls.2023.1304327

**Published:** 2024-01-17

**Authors:** Andrea Pagano, Carolina Gomes, Evy Timmerman, Paweł Sulima, Jerzy Andrzej Przyborowski, Dariusz Kruszka, Francis Impens, Jorge Almiro Pinto Paiva

**Affiliations:** ^1^ Department of Integrative Plant Biology, Institute of Plant Genetics, Polish Academy of Sciences, Poznań, Poland; ^2^ VIB-UGent Center for Medical Biotechnology, Ghent, Belgium; ^3^ Department of Biomolecular Medicine, Ghent University, Ghent, Belgium; ^4^ VIB Proteomics Core, Ghent, Belgium; ^5^ Department of Genetics, Plant Breeding and Bioresource Engineering, University of Warmia and Mazury in Olsztyn, Olsztyn, Poland

**Keywords:** purple willow, cytidine analogue, DNA methylation, plant development, proteomic

## Abstract

**Introduction:**

DNA methylation plays major roles in the epigenetic regulation of gene expression, transposon and transcriptional silencing, and DNA repair, with implications in developmental processes and phenotypic plasticity. Relevantly for woody species, DNA methylation constitutes a regulative layer in cell wall dynamics associated with xylogenesis. The use of methyltransferase and/or demethylase inhibitors has been proven informative to shed light on the methylome dynamics behind the regulation of these processes.

**Methods:**

The present work employs the cytidine analog zebularine to inhibit DNA methyltransferases and induce DNA hypomethylation in *Salix purpurea* plantlets grown *in vitro* and in soil. An integrative approach was adopted to highlight the effects of zebularine on proteomic dynamics, revealing age-specific (3 weeks of *in vitro* culture and 1 month of growth in soil) and tissue-specific (stem and root) effects.

**Results and discussion:**

After 3 weeks of recovery from zebularine treatment, a decrease of 5-mC levels was observed in different genomic contexts in the roots of explants that were exposed to zebularine, whereas a functionally heterogeneous subset of protein entries was differentially accumulated in stem samples, including entries related to cell wall biosynthesis, tissue morphogenesis, and hormonal regulation. Significant proteomic remodeling was revealed in the development from *in vitro* to in-soil culture, but no significant changes in 5-mC levels were observed. The identification of tissue-specific proteomic hallmarks in combination with hypomethylating agents provides new insights into the role of DNA methylation and proteome in early plant development in willow species. Proteomic data are available *via* ProteomeXchange with identifier PXD045653. WGBS data are available under BioProject accession PRJNA889596.

## 1 Introduction

Along with histone modification, DNA methylation represents a pivotal determinant of chromatin organization and epigenetic regulation. It is involved in a wide variety of processes such as gene expression regulation, transposon silencing, DNA repair, stress memory, and trait inheritance (Begcy and Dresselhaus, 2018; Zhang et al., 2018; Kim, 2019). In plants, methylation in cytosine position 5′ (5-mC) occurs in different sequence contexts, such as CG dinucleotides, CHG and CHH trinucleotides (where H represents A, T, or C), and genomic regions, such as at the promoters, at intron–exon junctions, or within transcribed sequences (Kumar et al., 2018). Particularly, methylation occurring on the gene promoters has been associated with transcriptional silencing, as it is able to alter the binding efficiency of transcriptional factors or to enhance the binding of transcriptional repressor, although contrary examples have been also reported in *Arabidopsis thaliana* and *Solanum lycopersicum* (Zhang et al., 2018; Zhao et al., 2019).

Epigenetic signatures modulate development, adaptive processes, and phenotypic plasticity, thus expanding the scope of genetic diversity. Specific methylation patterns have been correlated with morphological traits occurring under different environmental conditions such as temperature (e.g., in *Ranunculus kuepferi*, Syngelaki et al., 2020, and in *Populus nigra*, Vanden Broeck et al., 2018), field *versus* garden growth (e.g., in *Scabiosa columbaria*, Groot et al., 2018), and soil salinity (e.g., in *Phoenix dactylifera*, Al-Harrasi et al., 2018). Moreover, a dynamic interplay exists between DNA methylation, nuclear proteome, and stress memory mechanisms, as highlighted in heat-stressed *Pinus radiata* seedlings (Lamelas et al., 2020), whereas the hypomethylation of 5mC was associated with bud sprouting in *Paeonia suffruticosa* in response to temperature changes (Xin et al., 2019). Relevantly for adaptation to seasonal dynamics, the transition from dormant to developing cambium in *Populus tomentosa* has been associated with changes in DNA methylation and gene expression (Chen et al., 2021). Considering arboreal species, DNA methylation dynamics are reportedly involved into developmental processes such as the regulation of bud dormancy, acclimation, and biomass production (e.g., in *Populus* × *euramericana*, Le Gac et al., 2018). More specifically, differential DNA methylation is involved into cell wall development, cell division, and cell expansion in several woody species, including *Populus trichocarpa* (Zhang et al., 2020), *Populus deltoides × P. euramericana* (Luo et al., 2021), and *Populus tomentosa* (Chen et al., 2021). Compatibly, the inhibition of DNA methylation by 5-azacytidine resulted into impaired cell wall synthesis, as shown in, e.g., in *Phyllostachys edulis* (Liufu et al., 2023).

A wide range of enzymes, namely, methyltransferases and demethylases, are involved in the control of DNA methylation levels, thus playing fundamental roles in these processes. In this concern, the use of specific chemicals able to interfere with DNA methylation has been proven informative to unveil the role of methylation on transcriptional regulation and plant development (Chang and Pikaard, 2005; Griffin et al., 2016). Exposure of *Arabidopsis* apices to zebularine, a cytidine analog and inhibitor of DNA methyltransferases, resulted in the differential regulation of key DNA repair genes (Liu et al., 2015), whereas the use of another methyltransferase inhibitor, 5-azacytidine, resulted in the demethylation and differential expression of multiple genes encoding transcriptional factors in citrus *calli* (Xu et al., 2017). In addition to the use of methyltransferase inhibitors, further approaches have been employed to study the mechanisms and implications of DNA methylation. In this concern, the availability of multigenerational epimutants (mutants in the DNA methylation patterns) has unveiled the relevance of methylation and epimutation in plant inheritance, with effects on transcriptomic dynamics (Chodavarapu et al., 2012), whereas the heterologous expression of *Salix viminalis* methyl-CpG-binding domain protein is able to regulate the methylation and the expression of several genes involved in flowering in *Arabidopsis thaliana* (Cheng et al., 2020).

Despite the widely reported influence of DNA methylation on gene expression, few studies assessed the interplay between DNA methylation and proteomic changes, especially considering woody species used for biomass production. For example, proteomic changes, along with modifications of growth and embryogenic potential, have been observed in somatic hybrid larch (*Larix* × *eurolepis*) embryos during maturation in the presence of hypomethylating agents (5-azacytidine) and hypermethylating agents (hydroxyurea) (Teyssier et al., 2014).

In addition to their historical use as sources of pharmaceutically relevant compounds, *Salix* spp. are being evaluated as bioremediation and carbon sequestration tools and as biomass sources for biofuel production (Agostini et al., 2015). The recent literature reports proteomic studies in few willow species. Differential responses have been highlighted in the leaf and root proteome of *Salix viminalis* in the presence of soil contaminants, such as chromium (Zemleduch-Barylska and Lorenc-Plucińska, 2016). The response to heavy metal soil contaminants at the level of the proteome has been studied also in *Salix fragilis* and *Salix aurita* (Evlard et al., 2014a, Evlard et al., 2014b), providing new insights for the selection of varieties and clones suitable for bioremediation strategies. Willow species have been relevant also for testing the effectiveness of soil improvement strategies. For instance, differences in the activation of defense and signaling pathways were highlighted at the proteomic level in *Salix viminalis* grown in metal-contaminated soil and amended soil, whereas studying both root and stem proteome provided precious information concerning contaminant translocation and systemic responses (Lebrun et al., 2020). The effects of cadmium pollution were studied in *Salix matsudana* in terms of xylem morphology, cell wall thickening, and changes in transcriptome and proteome, with the upregulation of phenylpropanoid and lignin biosynthesis pathways (Yu et al., 2023). Wood formation represents another topical aspect concerning willow species. Xylogenesis is a complex and regulated developmental process studied in several tree species and with economic relevance for pulp and timber production, in addition to the aforementioned applications for biofuel production and carbon sequestration (Mellerowicz et al., 2001; Agostini et al., 2015; Wang et al., 2016). Differential DNA methylation has been associated with wood differentiation (Wang et al., 2016) and with seasonal cambium development (Chen et al., 2021) in *Populus tomentosa*. Nonetheless, to date, scarce information is available on the relationship between DNA methylation and proteomic dynamics in *Salix* spp. Given the widely reported roles of methylation in plant development, tissue differentiation, and response to environmental stimuli, this study assumes the hypothesis that altered DNA methylation levels can induce changes in protein accumulation profiles, highlighting protein entries that can act as molecular hallmarks of the processes regulated or influenced by DNA methylation. Therefore, the aims of this study are 1) to provide a novel insight on the effects of a hypomethylating agent, namely, zebularine, on the proteome of *Salix purpurea* stems and roots and 2) to assess the proteome dynamics of *Salix purpurea* in different growing environments in *in vitro* explants and plants potted in soil. The resulting information aimed at shedding new light on the processes underlying phenotypic plasticity in willow and other woody species.

## 2 Materials and methods

### 2.1 Plant material

*S. purpurea in vitro* plantlets were obtained by bud culture of the PSP33 genotype selected from the *Salix purpurea* germplasm collection at the University Warmia Mazury in Olsztyn (Poland) (Sulima et al., 2017a, 2017b, 2017c). The conditions for *in vitro* tissue culture were previously optimized for *S. purpurea* genotypes (Gomes et al., 2019). Namely, for the present work, actively growing stems of 5 mm–10 mm diameter were cut into nodal segments of 5 mm–10 mm length, each one containing one or two axillary buds. The resulting explants were surface sterilized for 15 min in a 10% (v/v) sodium hypochlorite solution and subsequently rinsed three times with sterile water. The explants were then collocated in an upright position in jars containing full-strength MS30 medium (Murashige and Skoog, Sigma-Aldrich, St. Louis, USA), containing 30 g L^−1^ sucrose (Duchefa, The Netherlands) and solidified with 8 g L^−1^ agar (Micro Agar, Duchefa, The Netherlands). The *in vitro* explants were kept under cool white fluorescent light at 270 μmol m^−2^ s^−1^, 16-h light/8-h dark photoperiod, 40% humidity, and day/night temperature of 20°C–22°C. After 3–4 weeks, 10–15-mm plantlets were excised from the stem and subcultured to fresh MS30. To maintain and propagate the *S. purpurea in vitro* culture, 15 mm–20 mm explants (with two axillary buds) were subcultured to fresh MS30 every 6 weeks.

### 2.2 Treatment administration

*S. purpurea in vitro* apical explants (15 mm–20 mm) were transferred to glass jars containing full-strength MS30 medium and grown under the same culture conditions described in the previous section. *In vitro* culturing was carried out in cylinder jars 12 cm in height and 6 cm in diameter, containing approximately 50 mL of culture media and four plantlets per jar.

An experimental system was established as shown in **Supplementary Figure 1**. To administrate the treatments necessary to alter the 5-mC levels, the culture medium was supplemented with 25 µM and 50 µM zebularine (“ZEB25” and “ZEB50” plants, respectively), whereas no treatment was administered to control (“CTRL”) plants. Two zebularine concentrations were tested to highlight dose-dependent effects on plant growth and to select the most suitable concentration for subsequent analyses. The explants were grown under these conditions for 14 days. During this period of treatment, both zebularine doses prevented root formation. After treatment administration, apical explants (15 mm–20 mm) from treated and untreated plantlets were transferred to fresh MS30 medium non-supplemented with zebularine and grown for 21 days to allow recovery from the treatments and subsequent root development also in zebularine-treated samples. Stem (“S” samples) and root (“R” samples) tissue samples were collected after 21 days of recovery (“3W,” “three weeks” samples). After 21 days of recovery, the rooted plantlets were transferred into pots with soil, and grown in these conditions for 30 days (“1M,” “one month” samples). Growth in soil was carried out in square pots 11 cm × 11 cm × 12 cm. Soil was a mixture of peat and sand, produced on the basis of high peat deacidified with calcium carbonate, pH 5.5–6.5 (H_2_0), salt concentration below 0.4 g NaCl/dm^3^. The standard peat fraction ranged from 0 mm to 30 mm and met the requirements of the root systems of most plants species. Stem and root tissue samples were collected also after this period of culture in soil. All the samples were stored at −80°C until analysis.

### 2.3 Biometrical analysis

Plantlets were checked daily to monitor the development of root and stem growth and senescence progression over the duration of the experiment, during the treatment administration phase, and during the recovery phase. A minimum of 10 plantlets per experimental condition were used as biological replicates. Images were recorded for each plantlet and analyzed using ImageJ (https://imagej.net/ij/index.html) in order to measure the number and length of internodes and roots. Significant differences were statistically assessed by one-way ANOVA and Tukey’s test comparing CTRL, ZEB25, and ZEB50 samples.

### 2.4 DNA methylation analysis

In order to study the impact of zebularine on 5′C-demethylation of genome, whole-genome bisulfite sequencing (WGBS-seq) was used to identify methylated cytosines on a genome-wide scale and analyze the total variation of 5′C methylation. A total of 24 libraries were produced from biological triplicates of root and stem samples collected from plantlets treated with 0 and 50 µM zebularine (CTRL and ZEB50 samples, respectively), at two different stages: after 3 weeks of *in vitro* recovery (3W samples) and 1 month after transfer to the soil (1M samples). In order to obtain enough material for DNA extraction, pools of two plant samples were used for each replicate for samples after 3 weeks of recovery. DNA was isolated using CTAB (cetyltrimethylammonium bromide) buffer and 0.1% 2-mercaptoethanol, followed by three cycles of CIA (chloroform-isoamyl alcohol) extraction, isopropanol precipitation, and ethanol washing. The samples were treated with RNase A. DNA integrity was visually verified on agarose gel electrophoresis, and its purity was assessed spectrophotometrically using NanoDrop™ One Microvolume UV-Vis Spectrophotometer (Thermo Scientific, Waltham, MA, USA); the sample concentration was measured using the Qubit™ DNA BR Assay Kit (Invitrogen, Carlsbad, CA, USA). In order to identify methylated cytosines on a genome-wide scale, whole-genome sequencing of bisulfite-converted DNA was carried out and analyzed by Novogene (Beijing, China). At least 600 ng DNA per sample was used on WGBS library preparation. DNA was fragmented by sonication to 200 bp–400 bp with Covaris S220, followed by end repair and adenylation. Sonicated DNA fragments were ligated with cytosine-methylated barcodes and treated twice with bisulfite using the EZ DNA Methylation-Gold™ Kit (Zymo Research). The resulting single-strand DNA fragments were PCR amplificated using KAPA HiFi HotStart Uracil + ReadyMix (2X). The library concentration was quantified by the Qubit® 2.0 Fluorometer (Life Technologies, CA, USA) and quantitative PCR. The clustering of the index-coded samples was performed on a cBot Cluster Generation System using PE Cluster Kit cBot-HS (Illumina) according to the manufacturer’s instructions. After cluster generation, the library preparations were sequenced on an Illumina platform and 150-bp paired-end reads were generated. Image analysis and base calling were performed with the standard Illumina pipeline, and finally 150-bp paired-end reads were generated. Raw data were trimmed by Trimmomatic (Bolger et al., 2014). The Bismark software (version 0.12.5; Krueger and Andrews, 2011) was used to perform alignments of the bisulfite-treated reads to a reference genome with the default parameters. DNA methylation levels in the different genomic contexts were retrieved and were statistically analyzed on JASP using ANOVA to isolate the effects of treatment groups (CTRL *versus* ZEB50 samples), stage groups (3W *versus* 1M samples), and type of organ (R and S) and their interactions. *p*-Value effects lower than 0.05 were considered statistically significant. *Post-hoc* analysis was performed using Tukey and Duncan tests. WGBS data are available under BioProject accession PRJNA889596 (https://www.ncbi.nlm.nih.gov/bioproject/PRJNA889596).

### 2.5 Proteomic analysis

#### 2.5.1 Sample preparation

Shotgun proteomics analysis by LFQ-MS was used to study the impact of zebularine on proteome dynamics of stems and roots, at two developmental conditions. Whole-protein extractions were made from the roots and stem samples collected from plantlets treated with 0 µM and 50 µM zebularine (CTRL and ZEB50 samples, respectively), after 3 weeks of *in vitro* recovery (3W samples) and 1 month of after transfer to the soil (1M samples). A total of five to six biological replicates were considered for each combination of tissue, treatment, and timepoint. One plant was used for each replicate. The analysis was performed, for stems and roots in two parallel experiments. For each experiment, ~100 mg of frozen tissue was ground to a fine powder in liquid nitrogen. Powders were resuspended in 1 mL buffer (50 mM Tris pH 7.5) supplemented with a protease inhibitor (cOmplete Mini EDTA-free Protease inhibitor plates, Roche, one tablet for 12 mL buffer). Samples were vortexed, incubated on ice for 10 min, and centrifuged at 12,000 rpm for 10 min at 4°C. Supernatants were transferred to new tubes and stored at −80°C. Protein integrity was verified on polyacrylamide gel (TGX FastCast Acrylamide Solutions, Bio-Rad), and protein concentration was estimated using the Qubit™ Protein Assay Kit (Thermo Fisher Scientific). Dried protein extract was dissolved in 8 M urea and 20 mM HEPES pH 8.0 to a concentration of 1 µg/µL. Proteins were first reduced by addition of DTT to a concentration of 15 mM and incubation for 30 min at 55°C and then alkylated by addition iodoacetamide to a concentration of 10 mM and incubation for 15 min at room temperature in the dark. Samples were diluted with 20 mM HEPES pH 8.0 to a urea concentration of 4 M, and proteins were digested with 1 µg lysyl endopeptidase (Wako) (1/100, w/w) for 4 h at 37°C. Samples were further diluted with 20 mM HEPES pH 8.0 to a final urea concentration of 2 M, and proteins were digested with 1 µg trypsin (Promega) (1/100, w/w) overnight at 37°C. The resulting peptide mixture was purified using OMIX C18 pipette tips (Agilent) and eluted with 0.1% trifluoroacetic acid in water/acetonitrile, 40/60 (v/v). Peptides were dried completely by vacuum drying and stored at −20°C.

#### 2.5.2 LC-MS/MS analysis

Purified peptides were redissolved in MS solvent A (0.1% FA in water/ACN (98:2, v/v)) and injected for LC-MS/MS analysis on an UltiMate 3000 RSLCnano system in-line connected to an Orbitrap Fusion Lumos mass spectrometer (Thermo). Trapping was performed at 10 μL/min for 4 min in loading solvent A on a 20-mm trapping column (made in-house, 100-μm internal diameter (I.D.), 5-μm beads, C18 Reprosil-HD, Dr. Maisch, Germany). The peptides were separated on a 200-cm µPAC™ column (C18-endcapped functionality, 300-µm-wide channels, 5-µm porous-shell pillars, inter-pillar distance of 2.5 µm, and a depth of 20 µm; PharmaFluidics, Belgium). It was kept at a constant temperature of 50°C. Peptides were eluted by a linear gradient reaching 55% MS solvent B (0.1% formic acid in water/acetonitrile, 20:80 (v/v)) after 145 min and 99% MS solvent B at 150 min, followed by a 10-min wash at 99% MS solvent B and re-equilibration with MS solvent A (0.1% FA in water). The first 15 min, the flow rate was set to 750 nL/min, after which it was kept constant at 300 nL/min. The mass spectrometer was operated in data-dependent mode, automatically switching between MS and MS/MS acquisition. Full-scan MS spectra (300–1,500 m/z) were acquired in 3-s acquisition cycles at a resolution of 120,000 in the Orbitrap analyzer after accumulation to a target AGC value of 200,000 with a maximum injection time of 30 ms. The precursor ions were filtered for charge states (2–7 required), dynamic range (60 s; +/− 10 ppm window) and intensity (minimal intensity of 5E3). The precursor ions were selected in the multipole with an isolation window of 1.2 Da and accumulated to an AGC target of 12E3 or a maximum injection time of 40 ms and activated using HCD fragmentation (34% NCE). The fragments were analyzed in the ion trap analyzer at normal scan rate. The polydimethylcyclosiloxane background ion at 445.12003 Da was used for internal calibration (lock mass).

For each experiment, LC-MS/MS runs of all 23 samples were searched together using the MaxQuant algorithm (version 1.6.10.34) with default search settings, including a false discovery rate set at 1% at the PSM, peptide, and protein levels. Spectra were searched against the protein sequences in the *S. purpurea* databases (Spurpurea_289_v1.0.protein_primaryTranscriptOnly, containing 37.865 protein sequences and Spurpurea_289_v1, containing 61,520 protein sequences) both provided by Phytozome (https://phytozome-next.jgi.doe.gov/). The mass tolerance for precursor and fragment ions was set to 4.5 ppm and 20 ppm, respectively, during the main search. Enzyme specificity was set as the C-terminal to arginine and lysine, also allowing cleavage at proline bonds with a maximum of two missed cleavages. Variable modifications were set to oxidation of methionine residues and acetylation of protein N-termini, whereas carbamidomethylation on cysteine residues was set as fixed modification. Matching between runs was enabled with a matching time window of 0.7 min and an alignment time window of 20 min. Only proteins with at least one unique or razor peptide were retained, leading to the identification of 4,714 protein groups for root samples and 4,740 protein groups for stem samples. Proteins were quantified by the MaxLFQ algorithm integrated in the MaxQuant software. A minimum ratio count of two unique or razor peptides was required for quantification. Further data analysis was performed with the Perseus software (version 1.6.2.1) after loading the protein group file from MaxQuant. Reverse database hits and proteins that were only identified by site were removed, LFQ intensities were log_2_ transformed, and replicate samples were grouped. Proteins with less than three valid values in at least one group were removed, and missing values were imputed from a normal distribution around the detection limit leading to a list of 2,759 quantified protein groups for roots samples and 2,686 protein groups for stem samples that were used for further data analysis. Statistical analysis was performed on the log_2_ LFQ intensity values to identify the differentially accumulated protein entries across specific pairwise comparisons within the experimental system, highlighting the changes occurring with development (3 weeks of recovery *versus* 1 more month of growth in soil) and with the response to zebularine (CTRL samples *versus* ZEB50 samples) with the following criteria: two-tailed Student’s *t*-test was utilized for pairwise comparisons, with Benjamini–Hochberg false discovery rate (FDR) adjusted *p*-value considered significant when <0.05, with correction coefficient S_o_ = 1.

The mass spectrometry proteomic data have been deposited to the ProteomeXchange Consortium *via* the PRIDE (Perez-Riverol et al., 2022) partner repository with the dataset identifier PXD045653.

### 2.6 Bioinformatic analysis

*Salix purpurea* genome version V1.0 publicly available on the Phytozome database (https://phytozome.jgi.doe.gov/pz/portal.html#!info?alias=Org_Spurpurea) was utilized as a reference genome for this study to analyze methylomics and proteomics data. AgriGO v2.0 (http://systemsbiology.cau.edu.cn/agriGOv2/) was used to obtain the GO terms enriched in the developmental transition (from 3 weeks of *in vitro* recovery to another month of growth in soil), using the default settings with 5 as the minimum number of mapping entries per GO term. The retrieved GO terms (particularly biological process) were subsequently uploaded into ReviGO (http://revigo.irb.hr/) to graphically represent the GO term plots and their interaction networks (Supek et al., 2011), using the default settings with the small (0.5) cutoff to select the hierarchically highest GO terms to be displayed in the networks. The Phytozome BioMar #146; tool (https://phytozome-next.jgi.doe.gov/) was used to characterize the differentially accumulated protein entries. Venn diagrams were obtained using Venny2.0 (Oliveros, 2007) (https://bioinfogp.cnb.csic.es/tools/venny/).

## 3 Results

### 3.1 Zebularine inhibits root formation and stem growth

**Figure 1:**
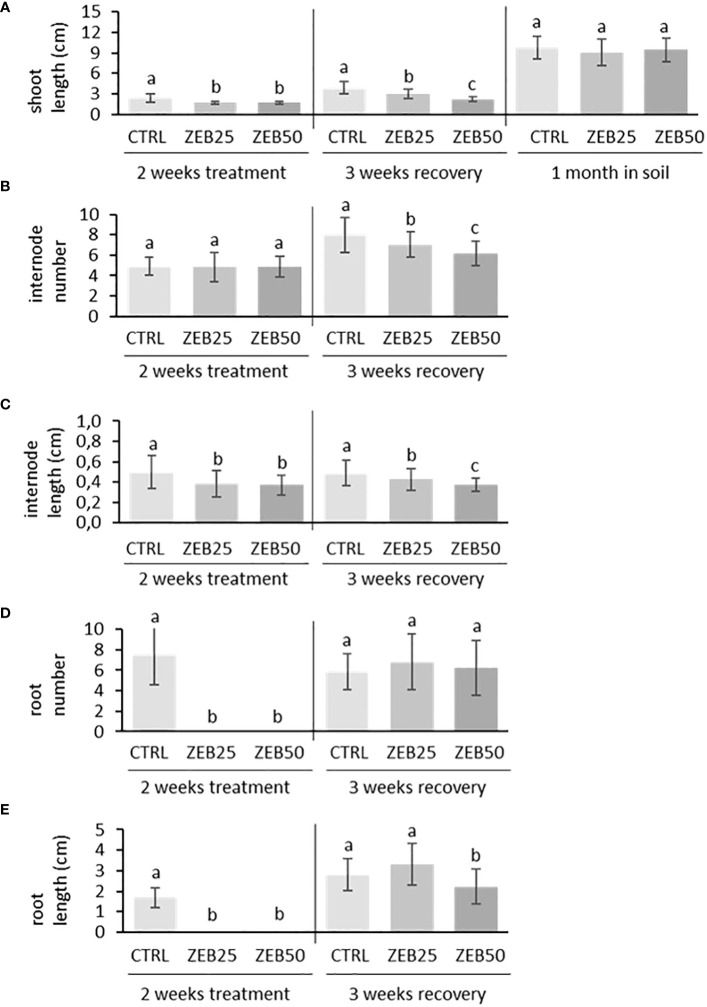
Biometrical response of *S. purpurea* plantlets to zebularine doses after 2 weeks of treatment, after 3 weeks of *in vitro* recovery, and after another month of growth in soil. **(A)** Stem length after 2 weeks of treatment, after 3 weeks of *in vitro* recovery, and after another month of growth in soil. **(B)** Average number of internodes per plant after 2 weeks of treatment and 3 weeks of *in vitro* recovery. **(C)** Internode length after 2 weeks of treatment and 3 weeks of *in vitro* recovery. **(D)** Average number of roots per plant after 2 weeks of treatment and 3 weeks of *in vitro* recovery. **(E)** Root length after 2 weeks of treatment and 3 weeks of *in vitro* recovery. CTRL, control; ZEB25, 25 µM zebularine; ZEB50, 50 µM zebularine. Means without a common superscript letter are significantly different (*p*-val. < 0.05) as analyzed by one-way ANOVA and Tukey test comparing CTRL, ZEB25, and ZEB50.

In order to evaluate the effects of demethylation on plant development, *in vitro* apical explants were grown in media supplemented with the demethylating agent zebularine (25 µM and 50 µM) for 14 days of treatment. They were subsequently transferred to fresh non-supplemented media for 21 days of recovery and then to soil for another month. As shown in **Figure 1**, apical explants cultured in media supplemented with both concentrations of zebularine did not develop roots throughout the 2 weeks of treatment administration, whereas root formation was not suppressed in control explants. After 2 weeks of treatment, explants displayed reduced stem length and reduced internode length in response to both doses of zebularine in comparison with control explants. After 3 weeks of recovery in non-supplemented media, the impairment of aerial part development was still detectable in plantlets that had been exposed to zebularine, in terms of reduced stem length, reduced internode length, and reduced internode number. Zebularine-treated plants developed roots only after transferring to non-supplemented media, displaying impaired root development after 3 weeks of recovery in response to the higher zebularine dose. After 3 weeks of *in vitro* recovery, plantlets were transferred to soil. After 1 month of growth in soil, no significant differences in terms of stem length were observed in response to zebularine. Globally, the higher zebularine concentration (50 μM) resulted in more contrastive phenotypes and was therefore selected for subsequent analyses.

### 3.2 Zebularine reduces DNA methylation levels in *S. purpurea* stems and roots after 3 weeks of *in vitro* recovery in zebularine-free media

To assess the total DNA methylation levels, specifically the percentage of 5-methylcytosine (5-mC) in each methylation context, a total of 24 high-quality WGBS libraries was produced and sequenced, for control and treated stems and root samples collected from *in vitro* and potted plant samples. A total of 381.5 Gb of raw bases, with an average 15.22 Gb of clean reads per library and an average bisulfite conversion rate of 99.66% per library, was obtained (**Supplementary Table 1**). The different sequence contexts in which 5′-cytosine methylation can occur in each sample were also analyzed. The average values for the all the samples analyzed were 6.6%, 29.9%, 14.9%, and 1.7% for mC (isolated cytosine), mCpG dinucleotides (cytosine–phosphate–guanine sites), mCHG trinucleotides, and mCHH trinucleotides (where H represents adenosine, cytidine, or thymidine), respectively (**Supplementary Table 1**).

The treatment of zebularine induced a significant reduction on 5-mC in all contexts but for the CHH context (**Supplementary Data Sheet 1**). Significant reduction on 5-mC were also observed in roots when compared with stems in all contexts but for the CG context. For all 5-mC contexts, no significant changes on the level of 5-mC between *in vitro* and potted plants were detected. For the combination of time, developmental stage, and tissue, some significant differences on the methylation levels (*p*-value <0.05) were found: a) after the 3 weeks of recovery, a significant reduction on 5-mC levels in the C and CHG contexts were observed in stems of *in vitro* zebularine plants whereas for treated roots the reduction was only observed for the CHG context. b) After 1 month, for stems and roots, no significant changes on 5-mC levels were observed for all 5-mC contexts, when compared with treated and control plants.

### 3.3 Proteomic shifts in *S. purpurea* stems and roots recovering from zebularine exposure

Shotgun proteomics analysis by LFQ-MS was used to study the impact of zebularine on proteome dynamics of stems and roots, at two developmental conditions. Results data are available in **Supplementary Tables 2** and **3**. Excluding the contaminants and splicing variants from the identified protein groups, 4,688 proteins have been identified in stem samples and 4,668 in root samples, with 3,775 common entries; thus, 913 and 893 protein entries have been identified uniquely in stem and root samples, respectively (**Supplementary Figure 2**). Considering the hierarchically highest GO terms referring to biological processes, the majority of the identified proteins were ascribed to the broad categorizations such as metabolic and cellular processes, whereas localization, response to stimuli, regulation, and cellular component organization were also represented with more than 100 entries each. The contaminants that have been detected in stem samples, root samples, or both tissues were also assessed and are shown in **Supplementary Figure 3**.

*S. purpurea* stem and root proteomes were analyzed through pairwise comparisons to discern the effect of zebularine after 3 weeks of *in vitro* recovery and after another month of growth in soil, considering the effect of the treatment (comparing CTRL and ZEB50 samples) along with the developmental changes occurring between the *in vitro* and in-soil stages (comparing 3W and 1M samples).

**Figure 2:**
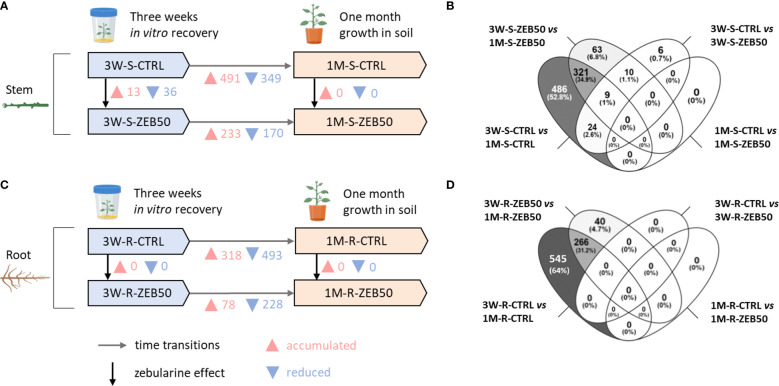
Overview of the differentially accumulated protein entries in *S. purpurea* proteome in response to 50 µM zebularine and in the transition from 3 weeks of recovery *in vitro* to 1 month growth in soil. **(A)** Differentially accumulated protein entries in stem samples. **(B)** Venn diagram outlining the degree of overlapping in the differentially accumulated protein entries in the pairwise comparisons involving stem samples. **(C)** Differentially accumulated protein entries in root samples. **(D)** Venn diagram outlining the degree of overlapping in the differentially accumulated protein entries in the pairwise comparisons involving root samples.

As shown in **Figure 2**, a strong proteome remodeling occurred between plants growing *in vitro* and plants subsequently grown in pots for a month. Considering the developmental stage effect, of 870 total protein entries differentially accumulated in control and zebularine-treated stems after 1 month of growth in soil, 321 were differentially accumulated in both conditions. For zebularine non-treated (CTRL) stems, 491 proteins were differentially accumulated and 349 proteins were significantly reduced after 1 month of growth in soil. The same tendency was observed for stems of plantlets that had been exposed to zebularine, with 233 protein entries differentially accumulated and 170 reduced after 1 month of growth in soil. Considering the response to zebularine after 3 weeks of *in vitro* recovery, 13 protein entries were accumulated and 36 reduced in stem samples, whereas no differentially accumulated protein entries were found in stems after another month of growth in soil. Concerning root samples, considering the total of 851 protein entries differentially accumulated in CTRL and ZEB50 roots after a month of growth in soil, 266 were differentially accumulated in both conditions. Considering control root samples, the majority of differentially accumulated protein entries were detected after 1 month of growth in soil, with 318 accumulated proteins and 493 reduced proteins. In the roots of plantlets that had been exposed to zebularine, 78 protein entries were accumulated and 228 reduced after 1 month of growth in soil. Nevertheless, no significant accumulation of proteins was observed in roots when comparing ZEB50 and CTRL samples after 3 weeks of *in vitro* recovery, or after another month of growth in soil.

### 3.4 Significant proteomic shifts are associated with *S. purpurea* stem and root development

The transition from 3-week recovery *in vitro* to 1-month growth in-soil in CTRL and ZEB50 stem and root samples has been considered to evaluate the persistence of the effects of zebularine exposure in stem and root development. GO term enrichment analysis was carried out considering the differentially accumulated proteins for each transition (3W-CTRL to 1M-CTRL, 3W-ZEB50 to 1M-ZEB50) in stem and root samples (**Supplementary Table 4**). As summarized in **Supplementary Figure 4**, broad biological process categories such as “metabolic processes,” “oxidation reduction,” “small molecule metabolic process,” and “carbohydrate metabolic process” were significantly enriched in developing *S. purpurea* stems and roots regardless of zebularine treatment. Other biological processes were more represented in stems than in roots regardless of zebularine exposure, including glucose, hexose, polysaccharide and monosaccharide catabolism, sulfur and sulfur compound metabolism, sulfur amino acid biosynthesis and metabolism, aminoglycan metabolism and catabolism process, chitin metabolism and catabolism, and cell wall macromolecule metabolism. Conversely, certain biological processes were more represented in root samples, including response to stimulus, translation, and ion transport. Globally, the development of stems and roots of plantlets that had been treated with zebularine displayed a reduced variety of significantly enriched biological processes (56 and 37 GO terms, respectively) than their control counterparts (79 and 46 GO terms, respectively).

**Figure 3:**
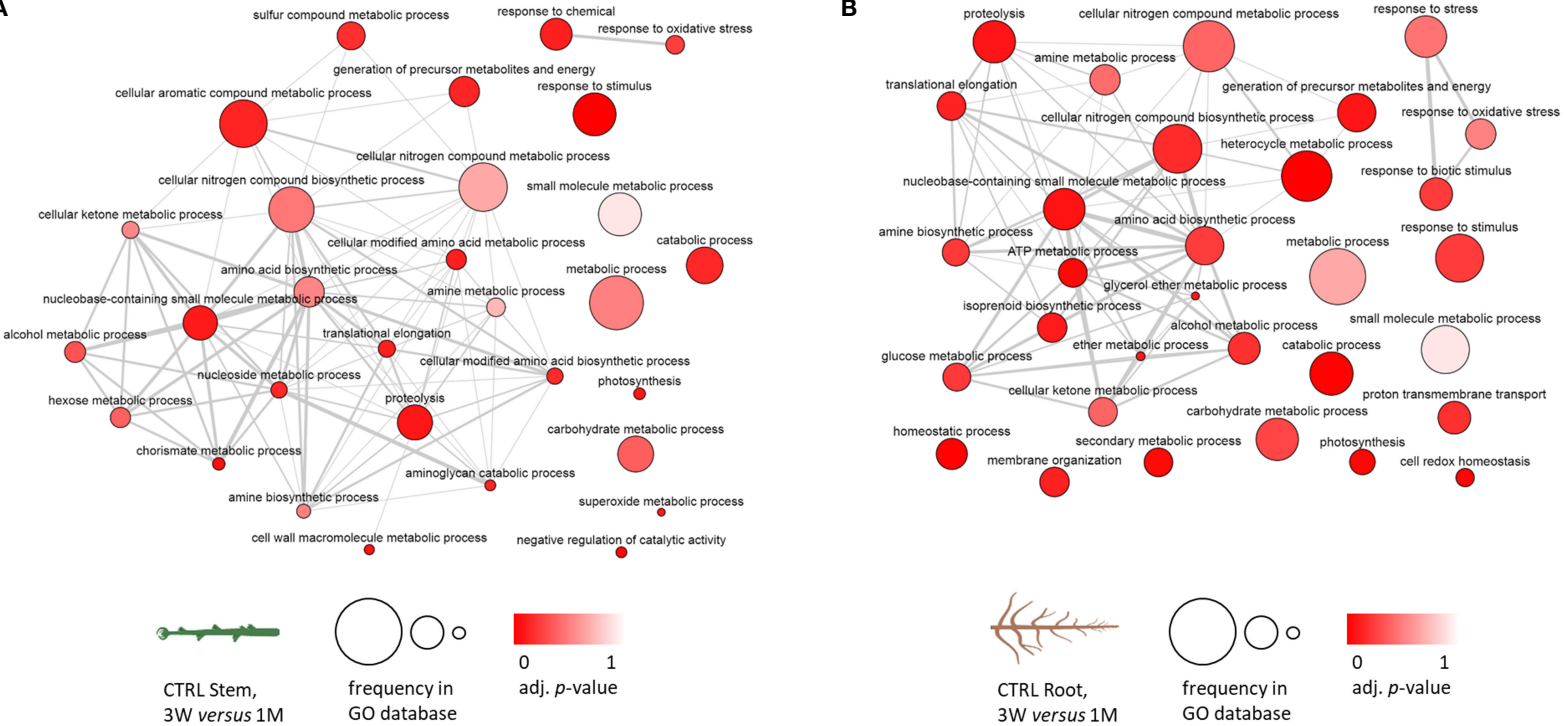
Biological process GO term analysis of the differentially accumulated protein entries in *S. purpurea* stem and root samples considering the transition between *in vitro* and in soil conditions in CTRL stems and roots; GO terms associated with five or more protein entries have been included. **(A)** Networks of the relations between GO terms in control stem samples. **(B)** Networks of the relations between GO terms in control root samples. The darker color of the bubble reflects a lower adjusted *p*-value obtained in the functional enrichment analysis, whereas the size of the bubble indicates the relative frequency of a given GO term in the GO annotation database. 3W, 3 weeks of *in vitro* growth; 1M, 1 month of growth in soil.

Starting from the GO term analysis of the differentially accumulated proteins in the developmental transition from 3 weeks of *in vitro* recovery to 1 month of growth in soil, GO term networks were obtained and set in order to highlight the hierarchically higher terms representing the biological processes (**Supplementary Figure 5**), molecular function (**Supplementary Figure 6**), and cellular component (**Supplementary Figure 7**). **Figure 3** reports the two networks that were obtained to assess the common and distinctive biological processes among stem and root control samples. As shown in Section 3.3, **Figure 2**, the fraction of proteins that were differentially accumulated in the transition from 3 weeks of recovery to 1 month is soil specifically in plants that had been exposed to zebularine, which was particularly low in both stems and roots. Subsequently, control samples were considered representative of the proteomic changes occurring in the developmental transition from *in vitro* to soil. A core cluster of hierarchically high biological processes was activated in both stem and root samples, including cellular nitrogen compound metabolic processes, amine and amino acid biosynthetic processes, proteolysis, cellular ketone metabolic processes, and also metabolic and catabolic processes, carbohydrate and small-molecule metabolic processes, response to stimulus, and photosynthesis, among others. A number of biological processes were represented only in stems, including cell wall macromolecule metabolic processes, negative regulation of catalytic activity, cellular modified amino acid biosynthetic process, chorismite metabolic process, sulfur compound metabolic process, and cellular aromatic compounds metabolic process, whereas other processes were represented only in roots, including glucose metabolic process, ATP metabolic process, proton transmembrane transport, membrane organization, isoprenoid biosynthetic process, secondary metabolite process, homeostasis, cell redox homeostasis, and response to stress.

### 3.5 Hallmarks of the response to zebularine in *S. purpurea* stem proteome

**Figure 4:**
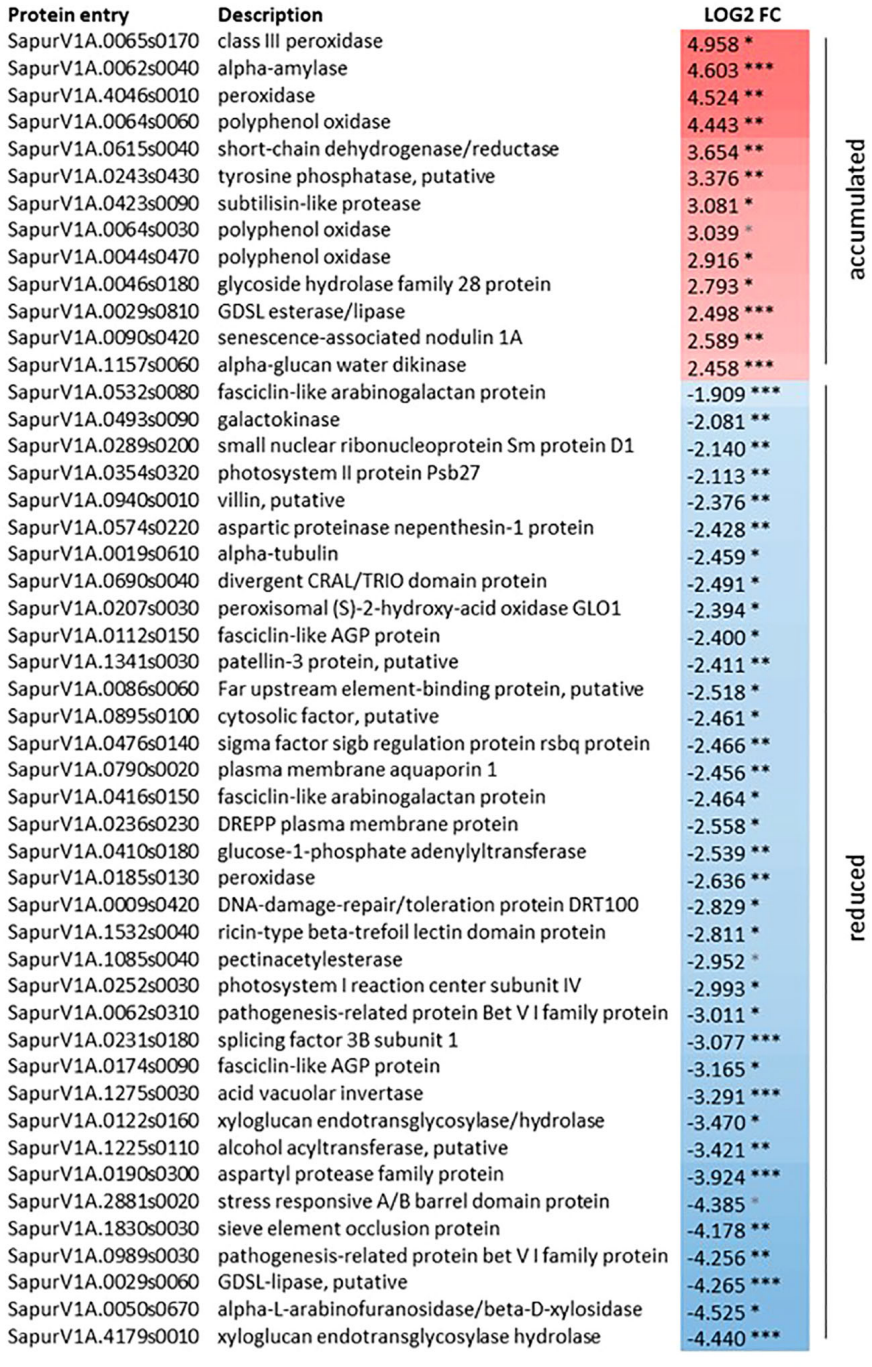
Differentially accumulated proteins in *S. purpurea* stems in response to zebularine after 3 weeks of *in vitro* recovery. *, adjusted *p*-value < 0.05; **, adjusted *p*-value < 0.01; ***, adjusted *p*-value < 0.001. Log_2_FC = LFQ_Zeb50S3W_ -LFQ_Zeb0S3W_.

Considering the effects of zebularine on *S. purpurea* stem and root proteome, pairwise comparisons have highlighted differentially accumulated proteins only in stem samples after 3 weeks of recovery, whereas no differentially accumulated proteins in response to zebularine were detected after another month of growth in soil. As shown in **Figure 4**, after the 3 weeks of recovery, 13 proteins were accumulated in treated plantlets and 36 proteins were accumulated in control plantlets. Two peroxidases (SapurV1A.0065s0170, SapurV1A.4046s0010), one alpha-amylase (SapurV1A.0062s0040), three polyphenol oxidases (SapurV1A.0064s0060, SapurV1A.0064s0030, SapurV1A.0044s0470), one subtilisin-like protease (SapurV1A0423s0090), and a senescence-associated nodulin 1A (SapurV1A0090s0420), among others, were identified among the upregulated proteins in treated stems.

The portion of downregulated proteins in treated stems after 3 weeks of recovery included entries that can be linked to xylogenesis, such as pectin acetylesterase (SapurV1A.1085s0040) and two xyloglucan endotransglycosylase hydrolase (SapurV1A.4179s0010, SapurV1A.0122s0160). A number of downregulated entries were associated with cell and/or tissue morphogenesis, sieve element occlusion protein (SapurV1A.1830s0030), and four fasciclin-like AGP proteins (SapurV1A.0112s0150, SapurV1A.0532s0080, SapurV1A.0416s0150, SapurV1A.0174s0090). Some other downregulated entries associated with cell and/or tissue morphogenesis were specifically linked to cytoskeleton and microtubule dynamics, namely, alpha tubulin (SapurV1A.0019s0610), DREPP plasma membrane protein (SapurV1A.0236s0230), and villin (SapurV1A.0940s0010). Proteins related to pigment metabolism and photosynthesis, such as photosystem I reaction center subunit IV (SapurV1A.0252s0030) and photosystem II protein Psb27 (SapurV1A.0354s0320), or DNA repair, such as a DNA-damage repair/toleration protein DRT100 (SapurV1A00009s0420), were also identified with a reduced accumulation in treated stems compared with the control ones.

As shown in **Supplementary Table 4**, the GO term enrichment analysis highlighted the biological processes represented among the differentially accumulated proteins and, in order to further reduce the complexity of the analysis, the significantly enriched GO terms were used into ReviGO to generate a hierarchical GO term network (**Supplementary Figure 8**). Considering the category of biological processes represented in the list of proteins that were differentially accumulated in response to zebularine in stems after 3 weeks of *in vitro* recovery, the main module was related with carbohydrate, polysaccharide, and lipid metabolism, dephosphorylation, and photosynthesis. Other modules included cytoskeleton organization and protein polymerization, microtubule-based processes, and response to abiotic and oxidative stress. Moreover, also considering molecular function and cellular component, the GO term analysis of the proteins differentially accumulated in zebularine-exposed samples corroborated the association with xyloglucan metabolism and cell wall and cytoskeleton dynamics.

## 4 Discussion

Purple willow is considered an ideal model species for bioenergy, due to its short-rotation coppice, small genome, and public availability of valuable genomic and genetic resources, including high-quality annotated reference genomes. Nevertheless, there is still a gap of knowledge concerning the dynamics of proteome and the role of DNA C-methylation during root and stem development of this species. The experimental system developed for this work aimed at providing novel insights on the roles of DNA methylation in root and stem development and proteome remodeling in the model woody species *S. purpurea*.

Artificially altered hypomethylated plants were produced by exposing the bottom of apical explants to the methyltransferase inhibitor zebularine for 2 weeks, followed by 3 weeks of recovery in zebularine-free medium. After the recovery period, the plants were potted and let to grow for 1 month. Unlike control explants, after 2 weeks of culture, the apical explants did not develop roots in the presence of 25 µM and 50 µM zebularine, but root formation recovered after 3 weeks in medium without zebularine. Thus, zebularine fully inhibited root formation and impaired stem growth as long as the apical explants were in contact with zebularine-supplemented media. Then, apical explants excised from control and zebularine-exposed plantlets were transplanted to zebularine-free media, and new roots developed from both control plantlets and plantlets that had been exposed to zebularine. After 3 weeks of *in vitro* recovery, considering plantlets that had been exposed to zebularine, stem and root growth was still displaying growth impairments. These results do not contradict the current literature, since a reduction of cell viability and growth was observed in *Vitis vinifera* cell cultures in response to comparable concentration ranges of zebularine (25 µM to 75 µM), in association to the upregulation of stress-related genes (Kong et al., 2022). Moreover, inhibition of stem bud regeneration was observed in *Petunia hybrida in vitro* cultures in the presence of the demethylating agents 5-azacytidine and 5-aza-2′-deoxycytidine (Prakash and Kumar, 1997). Reduction in root length has been previously reported in *Arabidopsis thaliana* in response to 10-µM zebularine treatment resulting in DNA damage and cell death in the apical meristem (Prochazkova et al., 2022). Consistently, the inhibition of DNA methylation by 5-azacytidine and RNA methylation by DZnepA in 5mC and m6A contexts altered lateral root development and impaired cell wall synthesis in *Phyllostachys edulis* (Liufu et al., 2023). However, azacytidine-induced hypomethylation has been shown to promote radicle formation in adult Arabidopsis explants but not in juvenile tissues (Massoumi et al., 2017). These contrasting results suggest that different molecular regulatory and defense mechanisms might be activated for different demethylation agents. A significant hypomethylation in the C and CHG contexts was detected in stems after the 3 weeks of recovery and in CHG in newly formed root samples after 3 weeks of recovery, whereas a comparable albeit not significant trend was detected in potted plants.

Together, these observations at the biometrical and methylome levels could be interpreted in terms of local effect of zebularine on DNA methylation levels and consequent inhibition/alteration of cell division and morphogenesis programs of the tissues in contact with this methylation agent (**Supplementary Figure 9**). Differentially accumulated proteins at the same recovery stage were detected only in stem samples, meaning that root morphogenesis was reestablished following a similar proteome program in treated and non-treated plants. In the stems of treated plants, a differential accumulation of proteins related to stress response was observed, along with a decrease of proteins involved in cell division, morphogenesis, and secondary cell wall remodeling, in accordance with the reduced length of stems and roots in zebularine-exposed plantlets compared with control ones. Among these downregulated proteins, four FASCICLIN-LIKE protein entries, a SIEVE ELEMENT OCCLUSION, and a VILLIN were identified. The differential cytosine methylation on FASCICLIN-LIKE AGP 13 has been linked to tissue differentiation in *Populus tomentosa* (Wang et al., 2016). The SIEVE ELEMENT OCCLUSION (SEO) proteins are particularly involved in phloem formation (Ernst et al., 2012). VILLIN proteins were reported to be involved in plant epidermis development, although further roles are ascribed to plant villins as actin-regulatory cytoskeletal components, including the elongation of pollen tubes and root hairs (Pei et al., 2012; Huang et al., 2015). Some of the downregulated entries associated with cell and tissue morphogenesis were linked to cytoskeleton and microtubule dynamics, namely, ALPHA TUBULIN, DREPP plasma membrane protein, and villin. The fundamental role of ALPHA TUBULIN in plant cell/tissue morphogenesis is widely reported (Gardiner, 2013), including more specific processes such as hairy root formation, relevantly for *in vitro* culture and tissue propagation (Zhang et al., 2021). The present study also reports a significant decrease of several proteins involved cell wall polysaccharide metabolic processes, such as two XYLOGLUCAN ENDO-TRANSGLYCOSYLASE HYDROLASE proteins. Although differential methylation was observed in root samples after 3 weeks of recovery, differentially accumulated proteins at the same stage were detected only in stem samples.

After 1 month of growth in soil, the assessed growth parameters and the methylation levels at the different contexts did not display significant differences in response to zebularine. Therefore, the demethylation effect of zebularine appears to be transitory. Nevertheless, a dilution effect cannot be discarded as the assessment of methylation levels from whole-tissue samples does not account for sub-tissue and single-cell differences in methylation levels. Another aspect that should be considered while evaluating the response to zebularine is the occurrence of side effects, e.g., in terms of toxicity. Based on the available data, the present work cannot exclude the contribution of zebularine-induced side effects, including DNA damage, on the observed growth impairments. In this concern, reduced root growth was observed in Arabidopsis in response to zebularine and in association with DNA damage and the activation of DNA repair pathways. Specifically, zebularine-induced DNA damage is associated with the formation of cross-links between MET1 and DNA during replication (Nowicka et al., 2020; Prochazkova et al., 2022; Dvořák Tomaštíková et al., 2023). On the other hand, the available literature highlights the efficiency of zebularine in inducing homogeneous and effective DNA hypomethylation with few experimental artifacts caused by cytotoxicity and a relatively long half-life compared with azacytidine (Cheng et al., 2003; Baubec et al., 2009). Previous studies in Arabidopsis report that 14 or 21 days of culture in non-supplemented media are sufficient for a nearly complete recovery from the growth impairments induced by a range of zebularine concentrations, whereas reduced levels of 5-mC were observed even after 8 weeks of recovery (Baubec et al., 2009).

In the present work, as expected, the bulk of significant differences in protein accumulation in roots and stems occurred along with the transfer from *in vitro* culture to the soil rather than as a consequence of the response to zebularine. Moreover, after 1 month of growth in soil, no significant differences in protein accumulation were observed in stems or roots between treated and non-treated plants. However, regardless of zebularine exposure, it was observed that the number of enriched processes of biological categories was higher in stem samples than in root samples. These results suggest that a more complex proteome remodeling, including carbohydrate metabolism and energy (glucose, hexose, polysaccharide, and monosaccharide catabolism), defense (chitin metabolism and catabolism), and cell wall macromolecule metabolism, occurred in the areal part of the plant in the transition to the new environment.

## 5 Conclusions

In this study, the proteome of *S. purpurea* was comprehensively assessed for the first time. Shotgun proteomics analysis by LFQ-MS was applied to stems and roots of plantlets grown *in vitro* and in soil pots. The data produced and made available represent a unique resource for this woody species that constitutes a model plant for bioenergy production. This study also investigated the effect of zebularine on methylation level, plant development, and proteome remodeling. Zebularine treatment was locally but transitorily effective on DNA demethylation of *in vitro* explants, with an inhibitory effect on plant development and growth, including the morphogenesis of adventitious roots. The obtained results are compatible with the hypothesis that the molecular machinery involved in cell division, morphogenesis, and secondary cell wall were challenged by zebularine, providing novel evidence to investigate the role of DNA methylation in the regulation of these processes. Further investigation is also necessary to assess the relative contribution of zebularine side effects and to establish specific causal relations between DNA methylation patterns, changes in gene expression, and differential protein accumulation. The results obtained by this study open new ways to the understanding of the mechanisms underlying the formation of adventitious roots and rejuvenation, with practical and technological implications not only for micropropagation strategies but also for technics of vegetative propagation of rooting-resilient woody plants (Mankessi et al., 2011; Wang et al., 2023). Studying the persistence and the effects of differential methylation patterns and how it affects gene expression and proteome dynamics is relevant to expanding the current knowledge of stress memory, phenotypic plasticity, and epigenetic inheritance (Kinoshita and Seki, 2014; Turgut-Kara et al., 2020; Miryeganeh, 2021; Inácio et al., 2022), including clonally propagated plants (Latzel et al., 2016).

## Data availability statement

The datasets presented in this study can be found in online repositories. The names of the repository/repositories and accession number(s) can be found below: BioProject accession PRJNA889596, and ProteomeXchange PXD045653.

## Author contributions

AP: Conceptualization, Formal analysis, Investigation, Writing – original draft, Writing – review & editing, Methodology, Resources. CG: Investigation, Resources, Writing – review & editing. ET: Data curation, Formal analysis, Investigation, Writing – review & editing, Methodology. PS: Writing – review & editing. JAP: Writing – review & editing. DK: Writing – review & editing. FI: Formal analysis, Methodology, Resources, Writing – review & editing, Conceptualization. JAPP: Conceptualization, Funding acquisition, Investigation, Methodology, Project administration, Resources, Supervision, Validation, Writing – original draft, Writing – review & editing.

## Supplementary material

Supplementary Data Sheet 1 ANOVA analysis results of the 5-mC levels on different methylation context.

Supplementary Table 1 Summary table of the results from WGBS analysis.

Supplementary Table 2 Proteomic data Stem samples.

Supplementary Table 3 Proteomic data Root samples.

Supplementary Table 4 Analyses of the GO Terms.

Supplementary Figure 1 Experimental system established for the present study and representative pictures of the resulting explants after two weeks of treatments and three weeks of *in vitro* recovery.

Supplementary Figure 2 Degree of overlapping between the proteins identified in stems and roots and most enriched GO terms (biological processes) among the proteins identified uniquely in stems, uniquely in roots, or in both tissues.

Supplementary Figure 3 Protein contaminants identified in stem and root samples.

Supplementary Figure 4 Biological process GO term analysis of the differentially accumulated protein entries in *S. purpurea* stem and root samples considering the transition between *in vitro* and in-soil conditions in CTRL and ZEB50 stems and roots, with asterisks indicating significantly enriched GO terms as provided by AgriGO. FRD. *, < 0.05; **, < 0.01; *** < 0.001.

Supplementary Figure 5 Networks of the relations between the hierarchically highest GO terms (Biological Process) identified among the differentially accumulated proteins in zebularine-treated/untreated stems and roots in the transition from three weeks of recovery and one more month of growth in soil. Circle size indicates size of the term, red color indicates lower FDR.

Supplementary Figure 6 Networks of the relations between the hierarchically highest GO terms (Molecular Function) identified among the differentially accumulated proteins in zebularine-treated/untreated stems and roots in the transition from three weeks of recovery and one more month of growth in soil. Circle size indicates size of the term, red color indicates lower FDR.

Supplementary Figure 7 Networks of the relations between the hierarchically highest GO terms (Cellular Component) identified among the differentially accumulated proteins in zebularine-treated/untreated stems and roots in the transition from three weeks of recovery and one more month of growth in soil. Circle size indicates size of the term, red color indicates lower FDR.

Supplementary Figure 8 Network of the relations between the hierarchically highest GO terms (Biological Process, Molecular Function, and Cellular Component) identified among the differentially accumulated proteins in stems after three weeks of recovery from zebularine. Circle size indicates size of the term, red color indicates lower *p*-value.

Supplementary Figure 9 Microscopy observations of roots and stems after three weeks of *in vitro* recovery, considering zebularine-treated and control plantlets.
