# Supplementary material for: Revealing the transitory and local effect of zebularine on development and on proteome dynamics of Salix purpurea
Source: Front Plant Sci. 2024 Jan 17;14:1304327. doi: 10.3389/fpls.2023.1304327 (PMC10827895; doi:10.3389/fpls.2023.1304327)
Supplement: Supplementary Data Sheet 1 — ANOVA analysis results of the 5-mC levels on different methylation context. [file DataSheet_1.pdf]

Results ANOVA - Zebularine effect on Different C-methylation contexts

ANOVA - Mean C

ANOVA - MeanC

Cases	Sum of Squares	df	Mean Square	F	p
Tissue	3.168	1	3.168	21.096	< .001
Treatment	3.953	1	3.953	26.320	< .001
Time	0.010	1	0.010	0.064	0.804
Tissue ✻ Treatment	0.016	1	0.016	0.107	0.748
Tissue ✻ Time	0.002	1	0.002	0.011	0.917
Treatment ✻ Time	0.687	1	0.687	4.573	0.048
Tissue ✻ Treatment ✻ Time	0.150	1	0.150	1.002	0.332
Residuals	2.403	16	0.150		

Note. Type III Sum of Squares

Post Hoc Tests

Standard

Post Hoc Comparisons - Tissue

		Mean Difference	SE	t	p_{tukey}
Internodes	Roots	0.727	0.158	4.593	< .001***

*** p < .001

Note. Results are averaged over the levels of: Treatment, Time

Post Hoc Comparisons - Treatment

		Mean Difference	SE	t	p_{tukey}
0 µM	50 µM	0.812	0.158	5.130	< .001***

*** p < .001

Note. Results are averaged over the levels of: Tissue, Time

Post Hoc Comparisons - Tissue ✻ Treatment

		Mean Difference	SE	t	p_{tukey}
Internodes 0 µM	Roots 0 µM	0.675	0.224	3.017	0.037*
	Internodes 50 µM	0.760	0.224	3.397	0.017*
	Roots 50 µM	1.538	0.224	6.875	< .001***
Roots 0 µM	Internodes 50 µM	0.085	0.224	0.380	0.981
	Roots 50 µM	0.863	0.224	3.859	0.007**
Internodes 50 µM	Roots 50 µM	0.778	0.224	3.479	0.015*

Note. Results are averaged over the levels of: Time

Note. P-value adjusted for comparing a family of 4

* p < .05, ** p < .01, *** p < .001

Post Hoc Comparisons - Time

		Mean Difference	SE	t	p_{tukey}
1 month potted	3 weeks recovery	−0.040	0.158	−0.253	0.804

Note. Results are averaged over the levels of: Tissue, Treatment

Post Hoc Comparisons - Tissue ✻ Time

		Mean Difference	SE	t	p_{tukey}
Internodes 1 month potted	Roots 1 month potted	0.710	0.224	3.173	0.027*
	Internodes 3 weeks recovery	−0.057	0.224	−0.253	0.994
	Roots 3 weeks recovery	0.687	0.224	3.069	0.033*
Roots 1 month potted	Internodes 3 weeks recovery	−0.767	0.224	−3.427	0.016*
	Roots 3 weeks recovery	−0.023	0.224	−0.104	1.000
Internodes 3 weeks recovery	Roots 3 weeks recovery	0.743	0.224	3.322	0.020*

* p < .05

Note. P-value adjusted for comparing a family of 4

Note. Results are averaged over the levels of: Treatment

Post Hoc Comparisons - Treatment ✻ Time

		Mean Difference	SE	t	p_{tukey}
0 μM 1 month potted	50 μM 1 month potted	0.473	0.224	2.116	0.190
	0 μM 3 weeks recovery	−0.378	0.224	−1.691	0.360
	50 μM 3 weeks recovery	0.772	0.224	3.449	0.016*
50 μM 1 month potted	0 μM 3 weeks recovery	−0.852	0.224	−3.806	0.008**
	50 μM 3 weeks recovery	0.298	0.224	1.333	0.556
0 μM 3 weeks recovery	50 μM 3 weeks recovery	1.150	0.224	5.140	< .001***

Note. Results are averaged over the levels of: Tissue

Note. P-value adjusted for comparing a family of 4

* p < .05, ** p < .01, *** p < .001

Post Hoc Comparisons - Tissue ✻ Treatment ✻ Time

		Mean Difference	SE	t	p_{tukey}
Internodes 0 µM 1 month potted	Roots 0 µM 1 month potted	0.500	0.316	1.580	0.755
	Internodes 50 µM 1 month potted	0.263	0.316	0.832	0.988
	Roots 50 µM 1 month potted	1.183	0.316	3.740	0.030*
	Internodes 0 µM 3 weeks recovery	−0.553	0.316	−1.749	0.659
	Roots 0 µM 3 weeks recovery	0.297	0.316	0.938	0.977
	Internodes 50 µM 3 weeks recovery	0.703	0.316	2.223	0.389
	Roots 50 µM 3 weeks recovery	1.340	0.316	4.235	0.011*
Roots 0 µM 1 month potted	Internodes 50 µM 1 month potted	−0.237	0.316	−0.748	0.994
	Roots 50 µM 1 month potted	0.683	0.316	2.160	0.422
	Internodes 0 µM 3 weeks recovery	−1.053	0.316	−3.329	0.064
	Roots 0 µM 3 weeks recovery	−0.203	0.316	−0.643	0.997
	Internodes 50 µM 3 weeks recovery	0.203	0.316	0.643	0.997
	Roots 50 µM 3 weeks recovery	0.840	0.316	2.655	0.206
Internodes 50 µM 1 month potted	Roots 50 µM 1 month potted	0.920	0.316	2.908	0.136
	Internodes 0 µM 3 weeks recovery	−0.817	0.316	−2.581	0.232
	Roots 0 µM 3 weeks recovery	0.033	0.316	0.105	1.000
	Internodes 50 µM 3 weeks recovery	0.440	0.316	1.391	0.849
	Roots 50 µM 3 weeks recovery	1.077	0.316	3.403	0.056
Roots 50 µM 1 month potted	Internodes 0 µM 3 weeks recovery	−1.737	0.316	−5.488	0.001**
	Roots 0 µM 3 weeks recovery	−0.887	0.316	−2.802	0.162
	Internodes 50 µM 3 weeks recovery	−0.480	0.316	−1.517	0.788
	Roots 50 µM 3 weeks recovery	0.157	0.316	0.495	1.000
Internodes 0 µM 3 weeks recovery	Roots 0 µM 3 weeks recovery	0.850	0.316	2.686	0.196
	Internodes 50 µM 3 weeks recovery	1.257	0.316	3.972	0.019*
	Roots 50 µM 3 weeks recovery	1.893	0.316	5.984	< .001***
Roots 0 µM 3 weeks recovery	Internodes 50 µM 3 weeks recovery	0.407	0.316	1.285	0.892
	Roots 50 µM 3 weeks recovery	1.043	0.316	3.297	0.068
Internodes 50 µM 3 weeks recovery	Roots 50 µM 3 weeks recovery	0.637	0.316	2.012	0.504

* p < .05, ** p < .01, *** p < .001

Note. P-value adjusted for comparing a family of 8

Dunnett

Dunnett Post Hoc Comparisons - Tissue

Comparison	Mean Difference	SE	t	$p_{dunnett}$
Roots - Internodes	−0.727	0.234	−3.107	0.005**

** p < .01

Note. Results based on uncorrected means.

Dunnett Post Hoc Comparisons - Treatment

Comparison	Mean Difference	SE	t	$p_{dunnett}$
50 μM - 0 μM	−0.812	0.221	−3.676	0.001**

** p < .01

Note. Results based on uncorrected means.

Dunnett Post Hoc Comparisons - Time

Comparison	Mean Difference	SE	t	$p_{dunnett}$
3 weeks recovery - 1 month potted	0.040	0.280	0.143	0.888

Note. Results based on uncorrected means.

ANOVA - Mean CG

ANOVA - MeanCG

Cases	Sum of Squares	df	Mean Square	F	p
Tissue	5.501	1	5.501	1.779	0.201
Treatment	23.900	1	23.900	7.728	0.013
Time	8.870	1	8.870	2.868	0.110
Tissue ✻ Treatment	0.473	1	0.473	0.153	0.701
Tissue ✻ Time	4.996	1	4.996	1.615	0.222
Treatment ✻ Time	2.300	1	2.300	0.744	0.401
Tissue ✻ Treatment ✻ Time	1.021	1	1.021	0.330	0.574
Residuals	49.483	16	3.093		

Note. Type III Sum of Squares

Post Hoc Tests

Standard

Post Hoc Comparisons - Tissue

		Mean Difference	SE	t	p_{tukey}
Internodes	Roots	0.958	0.718	1.334	0.201

Note. Results are averaged over the levels of: Treatment, Time

Post Hoc Comparisons - Treatment

		Mean Difference	SE	t	p_{tukey}
0 µM	50 µM	1.996	0.718	2.780	0.013*

* p < .05
Note. Results are averaged over the levels of: Tissue, Time

Post Hoc Comparisons - Tissue ✻ Treatment

		Mean Difference	SE	t	p_{tukey}
Internodes 0 µM	Roots 0 µM	0.677	1.015	0.666	0.908
	Internodes 50 µM	1.715	1.015	1.689	0.361
	Roots 50 µM	2.953	1.015	2.909	0.046*
Roots 0 µM	Internodes 50 µM	1.038	1.015	1.023	0.739
	Roots 50 µM	2.277	1.015	2.242	0.154
Internodes 50 µM	Roots 50 µM	1.238	1.015	1.220	0.624

* p < .05
Note. P-value adjusted for comparing a family of 4
Note. Results are averaged over the levels of: Time

Post Hoc Comparisons - Time

		Mean Difference	SE	t	p_{tukey}
1 month potted	3 weeks recovery	1.216	0.718	1.693	0.110

Note. Results are averaged over the levels of: Tissue, Treatment

Post Hoc Comparisons - Tissue ✻ Time

		Mean Difference	SE	t	p_{tukey}
Internodes 1 month potted	Roots 1 month potted	1.870	1.015	1.842	0.291
	Internodes 3 weeks recovery	2.128	1.015	2.096	0.196
	Roots 3 weeks recovery	2.173	1.015	2.141	0.183
Roots 1 month potted	Internodes 3 weeks recovery	0.258	1.015	0.254	0.994
	Roots 3 weeks recovery	0.303	1.015	0.299	0.990
Internodes 3 weeks recovery	Roots 3 weeks recovery	0.045	1.015	0.044	1.000

Note. P-value adjusted for comparing a family of 4
Note. Results are averaged over the levels of: Treatment

Post Hoc Comparisons - Treatment ✻ Time

		Mean Difference	SE	t	p_{tukey}
0 µM 1 month potted	50 µM 1 month potted	1.377	1.015	1.356	0.543
	0 µM 3 weeks recovery	0.597	1.015	0.588	0.934
	50 µM 3 weeks recovery	3.212	1.015	3.163	0.028*
50 µM 1 month potted	0 µM 3 weeks recovery	−0.780	1.015	−0.768	0.868
	50 µM 3 weeks recovery	1.835	1.015	1.807	0.306
0 µM 3 weeks recovery	50 µM 3 weeks recovery	2.615	1.015	2.576	0.085

* p < .05

Note. P-value adjusted for comparing a family of 4

Note. Results are averaged over the levels of: Tissue

Post Hoc Comparisons - Tissue ✻ Treatment ✻ Time

		Mean Difference	SE	t	p_{tukey}
Internodes 0 µM 1 month potted	Roots 0 µM 1 month potted	1.177	1.436	0.819	0.989
	Internodes 50 µM 1 month potted	0.683	1.436	0.476	1.000
	Roots 50 µM 1 month potted	3.247	1.436	2.261	0.369
	Internodes 0 µM 3 weeks recovery	1.097	1.436	0.764	0.993
	Roots 0 µM 3 weeks recovery	1.273	1.436	0.887	0.983
	Internodes 50 µM 3 weeks recovery	3.843	1.436	2.677	0.199
	Roots 50 µM 3 weeks recovery	3.757	1.436	2.616	0.219
Roots 0 µM 1 month potted	Internodes 50 µM 1 month potted	−0.493	1.436	−0.344	1.000
	Roots 50 µM 1 month potted	2.070	1.436	1.442	0.826
	Internodes 0 µM 3 weeks recovery	−0.080	1.436	−0.056	1.000
	Roots 0 µM 3 weeks recovery	0.097	1.436	0.067	1.000
	Internodes 50 µM 3 weeks recovery	2.667	1.436	1.857	0.595
	Roots 50 µM 3 weeks recovery	2.580	1.436	1.797	0.631
Internodes 50 µM 1 month potted	Roots 50 µM 1 month potted	2.563	1.436	1.785	0.638
	Internodes 0 µM 3 weeks recovery	0.413	1.436	0.288	1.000
	Roots 0 µM 3 weeks recovery	0.590	1.436	0.411	1.000
	Internodes 50 µM 3 weeks recovery	3.160	1.436	2.201	0.400
	Roots 50 µM 3 weeks recovery	3.073	1.436	2.140	0.432
Roots 50 µM 1 month potted	Internodes 0 µM 3 weeks recovery	−2.150	1.436	−1.497	0.798
	Roots 0 µM 3 weeks recovery	−1.973	1.436	−1.374	0.856
	Internodes 50 µM 3 weeks recovery	0.597	1.436	0.416	1.000
	Roots 50 µM 3 weeks recovery	0.510	1.436	0.355	1.000
Internodes 0 µM 3 weeks recovery	Roots 0 µM 3 weeks recovery	0.177	1.436	0.123	1.000
	Internodes 50 µM 3 weeks recovery	2.747	1.436	1.913	0.562
	Roots 50 µM 3 weeks recovery	2.660	1.436	1.852	0.598
Roots 0 µM 3 weeks recovery	Internodes 50 µM 3 weeks recovery	2.570	1.436	1.790	0.635
	Roots 50 µM 3 weeks recovery	2.483	1.436	1.729	0.670
Internodes 50 µM 3 weeks recovery	Roots 50 µM 3 weeks recovery	−0.087	1.436	−0.060	1.000

Note. P-value adjusted for comparing a family of 8

Dunnett

Dunnett Post Hoc Comparisons - Tissue

Comparison	Mean Difference	SE	t	$p_{dunnett}$
Roots - Internodes	−0.958	0.830	−1.153	0.261

Note. Results based on uncorrected means.

Dunnett Post Hoc Comparisons - Treatment

Comparison	Mean Difference	SE	t	$p_{dunnett}$
50 μM - 0 μM	−1.996	0.742	−2.690	0.013*

* p < .05

Note. Results based on uncorrected means.

Dunnett Post Hoc Comparisons - Time

Comparison	Mean Difference	SE	t	$p_{dunnett}$
3 weeks recovery - 1 month potted	−1.216	0.815	−1.492	0.150

Note. Results based on uncorrected means.

ANOVA - Mean CHG

ANOVA - MeanCHG

Cases	Sum of Squares	df	Mean Square	F	p
Tissue	17.340	1	17.340	13.053	0.002
Treatment	45.816	1	45.816	34.489	< .001
Time	0.928	1	0.928	0.699	0.416
Tissue ∗ Treatment	2.483	1	2.483	1.869	0.190
Tissue ∗ Time	1.441	1	1.441	1.084	0.313
Treatment ∗ Time	4.403	1	4.403	3.315	0.087
Tissue ∗ Treatment ∗ Time	1.771	1	1.771	1.333	0.265
Residuals	21.255	16	1.328		

Note. Type III Sum of Squares

Post Hoc Tests

Standard

Post Hoc Comparisons - Tissue

		Mean Difference	SE	t	p_{tukey}
Internodes	Roots	1.700	0.471	3.613	0.002**

** p < .01
Note. Results are averaged over the levels of: Treatment, Time

Post Hoc Comparisons - Treatment

		Mean Difference	SE	t	p_{tukey}
0 µM	50 µM	2.763	0.471	5.873	< .001***

*** p < .001
Note. Results are averaged over the levels of: Tissue, Time

Post Hoc Comparisons - Tissue ✻ Treatment

		Mean Difference	SE	t	p_{tukey}
Internodes 0 µM	Roots 0 µM	1.057	0.665	1.588	0.413
	Internodes 50 µM	2.120	0.665	3.186	0.027*
	Roots 50 µM	4.463	0.665	6.707	< .001***
Roots 0 µM	Internodes 50 µM	1.063	0.665	1.598	0.407
	Roots 50 µM	3.407	0.665	5.119	< .001***
Internodes 50 µM	Roots 50 µM	2.343	0.665	3.522	0.014*

* p < .05, ** p < .01, *** p < .001
Note. P-value adjusted for comparing a family of 4
Note. Results are averaged over the levels of: Time

Post Hoc Comparisons - Time

		Mean Difference	SE	t	p_{tukey}
1 month potted	3 weeks recovery	0.393	0.471	0.836	0.416

Note. Results are averaged over the levels of: Tissue, Treatment

Post Hoc Comparisons - Tissue ✻ Time

		Mean Difference	SE	t	p_{tukey}
Internodes 1 month potted	Roots 1 month potted	2.190	0.665	3.291	0.022*
	Internodes 3 weeks recovery	0.883	0.665	1.327	0.560
	Roots 3 weeks recovery	2.093	0.665	3.146	0.029*
Roots 1 month potted	Internodes 3 weeks recovery	−1.307	0.665	−1.964	0.242
	Roots 3 weeks recovery	−0.097	0.665	−0.145	0.999
Internodes 3 weeks recovery	Roots 3 weeks recovery	1.210	0.665	1.818	0.301

* p < .05

Note. P-value adjusted for comparing a family of 4

Note. Results are averaged over the levels of: Treatment

Post Hoc Comparisons - Treatment ✻ Time

		Mean Difference	SE	t	p_{tukey}
0 µM 1 month potted	50 µM 1 month potted	1.907	0.665	2.865	0.050*
	0 µM 3 weeks recovery	−0.463	0.665	−0.696	0.897
	50 µM 3 weeks recovery	3.157	0.665	4.744	0.001**
50 µM 1 month potted	0 µM 3 weeks recovery	−2.370	0.665	−3.562	0.012*
	50 µM 3 weeks recovery	1.250	0.665	1.878	0.276
0 µM 3 weeks recovery	50 µM 3 weeks recovery	3.620	0.665	5.440	< .001***

Note. Results are averaged over the levels of: Tissue

Note. P-value adjusted for comparing a family of 4

* p < .05, ** p < .01, *** p < .001

Post Hoc Comparisons - Tissue ✻ Treatment ✻ Time

		Mean Difference	SE	t	p_{tukey}
Internodes 0 μM 1 month potted	Roots 0 μM 1 month potted	1.003	0.941	1.066	0.955
	Internodes 50 μM 1 month potted	0.720	0.941	0.765	0.993
	Roots 50 μM 1 month potted	4.097	0.941	4.353	0.009**
	Internodes 0 μM 3 weeks recovery	−0.517	0.941	−0.549	0.999
	Roots 0 μM 3 weeks recovery	0.593	0.941	0.630	0.998
	Internodes 50 μM 3 weeks recovery	3.003	0.941	3.191	0.082
	Roots 50 μM 3 weeks recovery	4.313	0.941	4.583	0.006**
Roots 0 μM 1 month potted	Internodes 50 μM 1 month potted	−0.283	0.941	−0.301	1.000
	Roots 50 μM 1 month potted	3.093	0.941	3.287	0.069
	Internodes 0 μM 3 weeks recovery	−1.520	0.941	−1.615	0.736
	Roots 0 μM 3 weeks recovery	−0.410	0.941	−0.436	1.000
	Internodes 50 μM 3 weeks recovery	2.000	0.941	2.125	0.440
	Roots 50 μM 3 weeks recovery	3.310	0.941	3.517	0.045*
Internodes 50 μM 1 month potted	Roots 50 μM 1 month potted	3.377	0.941	3.588	0.039*
	Internodes 0 μM 3 weeks recovery	−1.237	0.941	−1.314	0.881
	Roots 0 μM 3 weeks recovery	−0.127	0.941	−0.135	1.000
	Internodes 50 μM 3 weeks recovery	2.283	0.941	2.426	0.293
	Roots 50 μM 3 weeks recovery	3.593	0.941	3.818	0.025*
Roots 50 μM 1 month potted	Internodes 0 μM 3 weeks recovery	−4.613	0.941	−4.902	0.003**
	Roots 0 μM 3 weeks recovery	−3.503	0.941	−3.723	0.031*
	Internodes 50 μM 3 weeks recovery	−1.093	0.941	−1.162	0.932
	Roots 50 μM 3 weeks recovery	0.217	0.941	0.230	1.000
Internodes 0 μM 3 weeks recovery	Roots 0 μM 3 weeks recovery	1.110	0.941	1.180	0.927
	Internodes 50 μM 3 weeks recovery	3.520	0.941	3.740	0.030*
	Roots 50 μM 3 weeks recovery	4.830	0.941	5.132	0.002**
Roots 0 μM 3 weeks recovery	Internodes 50 μM 3 weeks recovery	2.410	0.941	2.561	0.239
	Roots 50 μM 3 weeks recovery	3.720	0.941	3.953	0.020*
Internodes 50 μM 3 weeks recovery	Roots 50 μM 3 weeks recovery	1.310	0.941	1.392	0.848

* p < .05, ** p < .01

Note. P-value adjusted for comparing a family of 8

Dunnett

Dunnett Post Hoc Comparisons - Tissue

Comparison	Mean Difference	SE	t	$p_{dunnett}$
Roots - Internodes	−1.700	0.769	−2.210	0.038*

* p < .05

Note. Results based on uncorrected means.

Dunnett Post Hoc Comparisons - Treatment

Comparison	Mean Difference	SE	t	$p_{dunnett}$
50 µM - 0 µM	−2.763	0.613	−4.507	< .001***

*** p < .001

Note. Results based on uncorrected means.

Dunnett Post Hoc Comparisons - Time

Comparison	Mean Difference	SE	t	$p_{dunnett}$
3 weeks recovery - 1 month potted	−0.393	0.846	−0.465	0.647

Note. Results based on uncorrected means.

ANOVA MeanCHH

ANOVA - MeanCHH

Cases	Sum of Squares	df	Mean Square	F	p
Tissue	1.373	1	1.373	41.632	< .001
Treatment	0.336	1	0.336	10.192	0.006
Time	0.395	1	0.395	11.987	0.003
Tissue ∗ Treatment	0.035	1	0.035	1.069	0.316
Tissue ∗ Time	0.160	1	0.160	4.854	0.043
Treatment ∗ Time	0.205	1	0.205	6.227	0.024
Tissue ∗ Treatment ∗ Time	1.667×10^{-5}	1	1.667×10^{-5}	5.054×10^{-4}	0.982
Residuals	0.528	16	0.033		

Note. Type III Sum of Squares

Post Hoc Tests

Standard

Post Hoc Comparisons - Tissue

		Mean Difference	SE	t	p_{tukey}
Internodes	Roots	0.478	0.074	6.452	< .001***

*** p < .001
Note. Results are averaged over the levels of: Treatment, Time

Post Hoc Comparisons - Treatment

		Mean Difference	SE	t	p_{tukey}
0 μM	50 μM	0.237	0.074	3.192	0.006**

** p < .01
Note. Results are averaged over the levels of: Tissue, Time

Post Hoc Comparisons - Tissue ✻ Treatment

		Mean Difference	SE	t	p_{tukey}
Internodes 0 μM	Roots 0 μM	0.555	0.105	5.294	< .001***
	Internodes 50 μM	0.313	0.105	2.989	0.039*
	Roots 50 μM	0.715	0.105	6.820	< .001***
Roots 0 μM	Internodes 50 μM	−0.242	0.105	−2.305	0.138
	Roots 50 μM	0.160	0.105	1.526	0.446
Internodes 50 μM	Roots 50 μM	0.402	0.105	3.831	0.007**

* p < .05, ** p < .01, *** p < .001
Note. P-value adjusted for comparing a family of 4
Note. Results are averaged over the levels of: Time

Post Hoc Comparisons - Time

		Mean Difference	SE	t	p_{tukey}
1 month potted	3 weeks recovery	−0.257	0.074	−3.462	0.003**

** p < .01
Note. Results are averaged over the levels of: Tissue, Treatment

Post Hoc Comparisons - Tissue ✻ Time

		Mean Difference	SE	t	p_{tukey}
Internodes 1 month potted	Roots 1 month potted	0.315	0.105	3.005	0.038*
	Internodes 3 weeks recovery	−0.420	0.105	−4.006	0.005**
	Roots 3 weeks recovery	0.222	0.105	2.114	0.191
Roots 1 month potted	Internodes 3 weeks recovery	−0.735	0.105	−7.011	< .001***
	Roots 3 weeks recovery	−0.093	0.105	−0.890	0.810
Internodes 3 weeks recovery	Roots 3 weeks recovery	0.642	0.105	6.120	< .001***

* p < .05, ** p < .01, *** p < .001

Note. P-value adjusted for comparing a family of 4

Note. Results are averaged over the levels of: Treatment

Post Hoc Comparisons - Treatment ✻ Time

		Mean Difference	SE	t	p_{tukey}
0 µM 1 month potted	50 µM 1 month potted	0.052	0.105	0.493	0.960
	0 µM 3 weeks recovery	−0.442	0.105	−4.213	0.003**
	50 µM 3 weeks recovery	−0.020	0.105	−0.191	0.997
50 µM 1 month potted	0 µM 3 weeks recovery	−0.493	0.105	−4.706	0.001**
	50 µM 3 weeks recovery	−0.072	0.105	−0.684	0.902
0 µM 3 weeks recovery	50 µM 3 weeks recovery	0.422	0.105	4.022	0.005**

** p < .01

Note. P-value adjusted for comparing a family of 4

Note. Results are averaged over the levels of: Tissue

Post Hoc Comparisons - Tissue ✻ Treatment ✻ Time

		Mean Difference	SE	t	p_{tukey}
Internodes 0 μM 1 month potted	Roots 0 μM 1 month potted	0.390	0.148	2.630	0.214
	Internodes 50 μM 1 month potted	0.127	0.148	0.854	0.986
	Roots 50 μM 1 month potted	0.367	0.148	2.473	0.273
	Internodes 0 μM 3 weeks recovery	−0.607	0.148	−4.092	0.015*
	Roots 0 μM 3 weeks recovery	0.113	0.148	0.764	0.993
	Internodes 50 μM 3 weeks recovery	−0.107	0.148	−0.719	0.995
	Roots 50 μM 3 weeks recovery	0.457	0.148	3.080	0.101
Roots 0 μM 1 month potted	Internodes 50 μM 1 month potted	−0.263	0.148	−1.776	0.643
	Roots 50 μM 1 month potted	−0.023	0.148	−0.157	1.000
	Internodes 0 μM 3 weeks recovery	−0.997	0.148	−6.722	< .001***
	Roots 0 μM 3 weeks recovery	−0.277	0.148	−1.866	0.590
	Internodes 50 μM 3 weeks recovery	−0.497	0.148	−3.350	0.062
	Roots 50 μM 3 weeks recovery	0.067	0.148	0.450	1.000
Internodes 50 μM 1 month potted	Roots 50 μM 1 month potted	0.240	0.148	1.619	0.734
	Internodes 0 μM 3 weeks recovery	−0.733	0.148	−4.946	0.003**
	Roots 0 μM 3 weeks recovery	−0.013	0.148	−0.090	1.000
	Internodes 50 μM 3 weeks recovery	−0.233	0.148	−1.574	0.758
	Roots 50 μM 3 weeks recovery	0.330	0.148	2.226	0.387
Roots 50 μM 1 month potted	Internodes 0 μM 3 weeks recovery	−0.973	0.148	−6.565	< .001***
	Roots 0 μM 3 weeks recovery	−0.253	0.148	−1.709	0.683
	Internodes 50 μM 3 weeks recovery	−0.473	0.148	−3.192	0.082
	Roots 50 μM 3 weeks recovery	0.090	0.148	0.607	0.998
Internodes 0 μM 3 weeks recovery	Roots 0 μM 3 weeks recovery	0.720	0.148	4.856	0.003**
	Internodes 50 μM 3 weeks recovery	0.500	0.148	3.372	0.059
	Roots 50 μM 3 weeks recovery	1.063	0.148	7.172	< .001***
Roots 0 μM 3 weeks recovery	Internodes 50 μM 3 weeks recovery	−0.220	0.148	−1.484	0.805
	Roots 50 μM 3 weeks recovery	0.343	0.148	2.316	0.343
Internodes 50 μM 3 weeks recovery	Roots 50 μM 3 weeks recovery	0.563	0.148	3.799	0.026*

Note. P-value adjusted for comparing a family of 8

* p < .05, ** p < .01, *** p < .001

Dunnett

Dunnett Post Hoc Comparisons - Tissue

Comparison	Mean Difference	SE	t	$p_{dunnett}$
Roots - Internodes	−0.478	0.112	−4.266	< .001***

*** p < .001

Note. Results based on uncorrected means.

Dunnett Post Hoc Comparisons - Treatment

Comparison	Mean Difference	SE	t	$p_{dunnett}$
50 µM - 0 µM	−0.237	0.143	−1.656	0.112

Note. Results based on uncorrected means.

Dunnett Post Hoc Comparisons - Time

Comparison	Mean Difference	SE	t	$p_{dunnett}$
3 weeks recovery - 1 month potted	0.257	0.141	1.816	0.083

Note. Results based on uncorrected means.
