# Supplementary figures and images for: Revealing the transitory and local effect of zebularine on development and on proteome dynamics of Salix purpurea
Source: Front Plant Sci. 2024 Jan 17;14:1304327. doi: 10.3389/fpls.2023.1304327 (PMC10827895; doi:10.3389/fpls.2023.1304327)

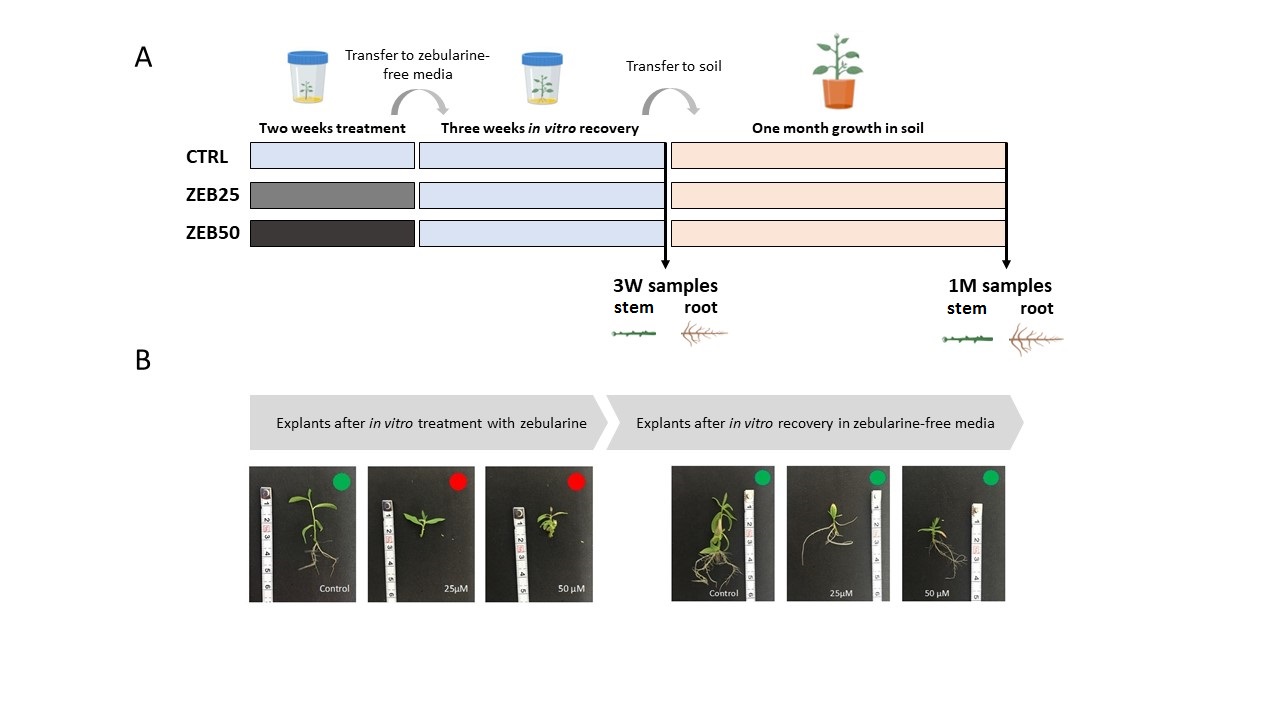

Supplement: Supplementary Figure 1 — Experimental system established for the present study and representative pictures of the resulting explants after two weeks of treatments and three weeks of in vitro recovery. [file Image_1.jpeg]

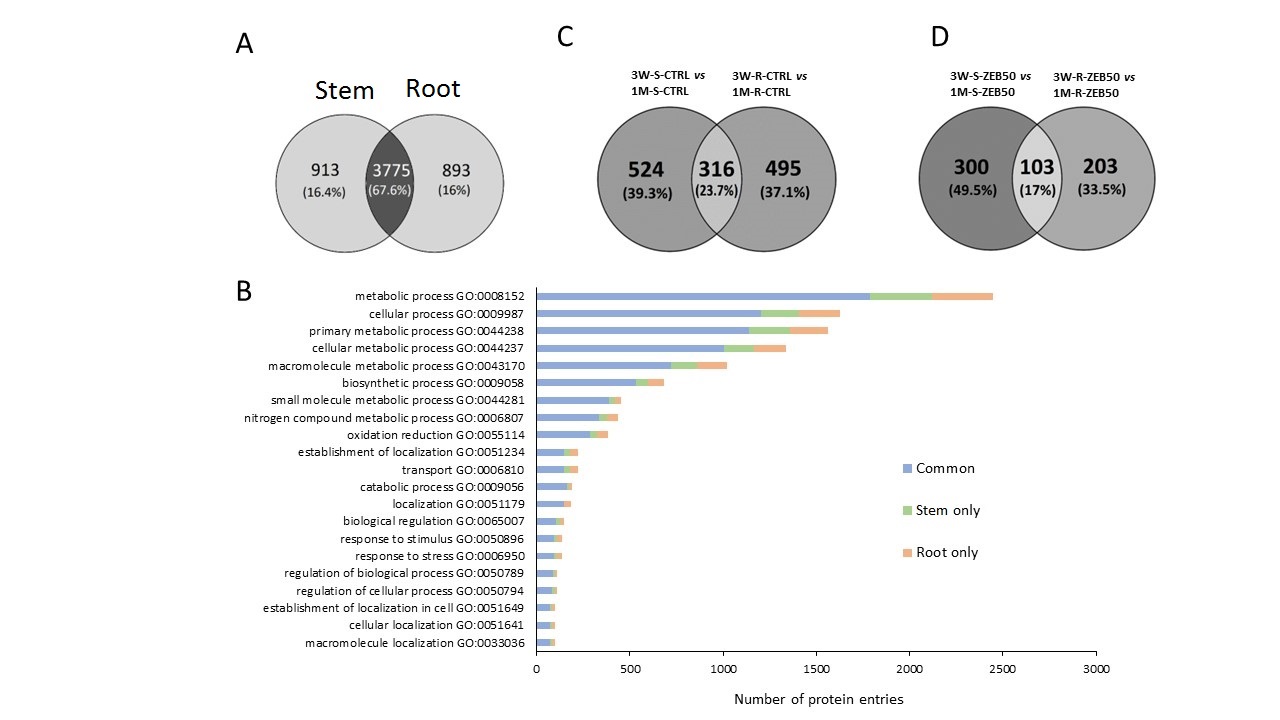

Supplement: Supplementary Figure 2 — Degree of overlapping between the proteins identified in stems and roots and most enriched GO terms (biological processes) among the proteins identified uniquely in stems, uniquely in roots, or in both tissues. [file Image_2.jpeg]

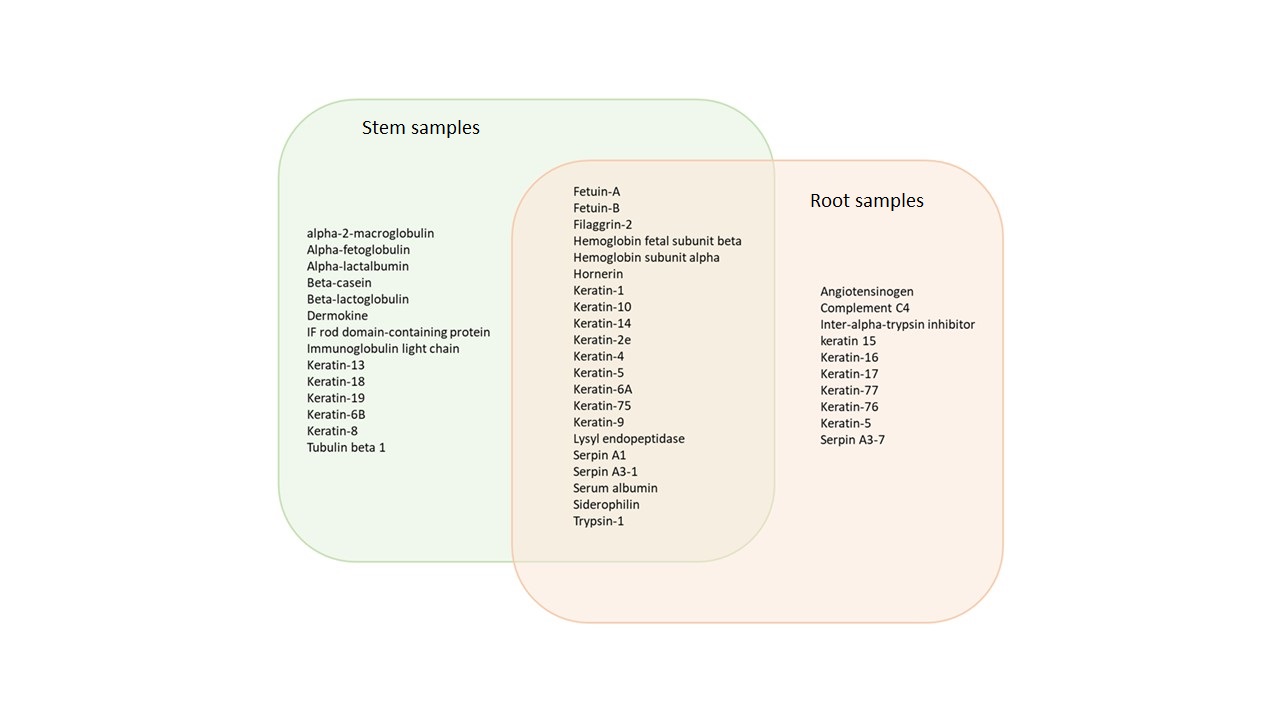

Supplement: Supplementary Figure 3 — Protein contaminants identified in stem and root samples. [file Image_3.jpeg]

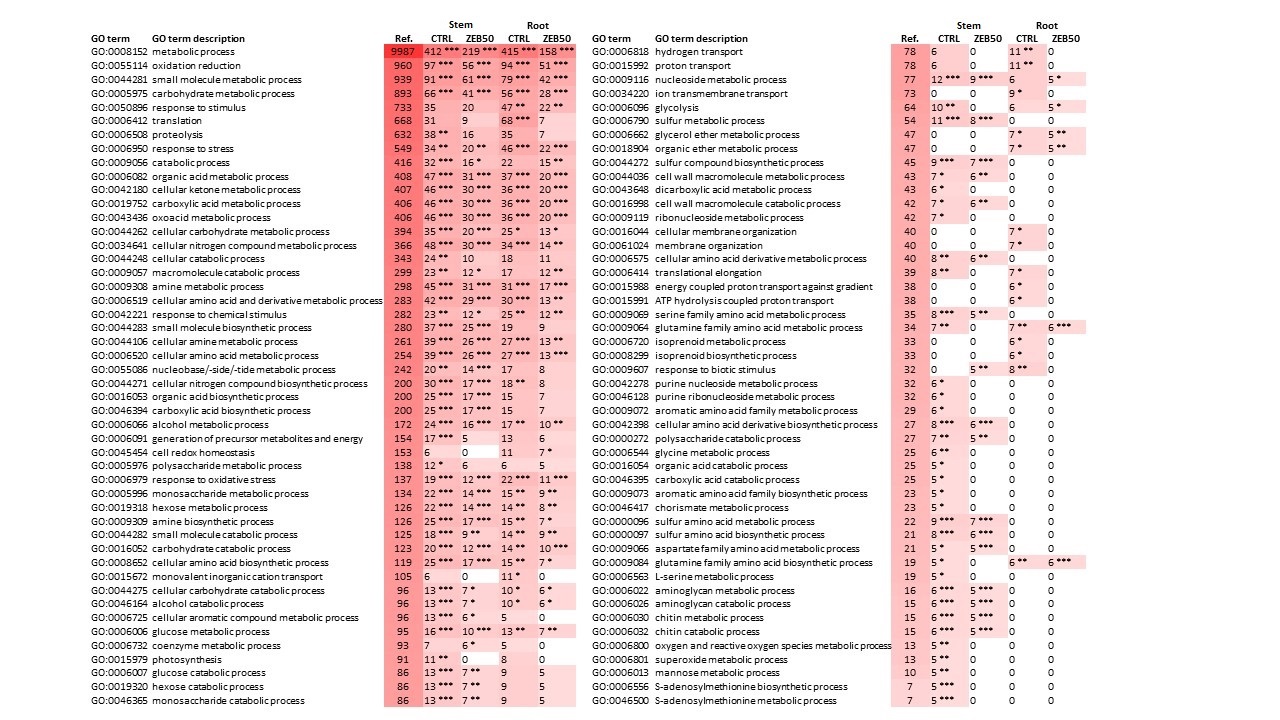

Supplement: Supplementary Figure 4 — Biological process GO term analysis of the differentially accumulated protein entries in S. purpurea stem and root samples considering the transition between in vitro and in-soil conditions in CTRL and ZEB50 stems and roots, with asterisks indicating significantly enriched GO terms as provided by AgriGO. FRD. *, < 0.05; **, < 0.01; *** < 0.001. [file Image_4.jpeg]

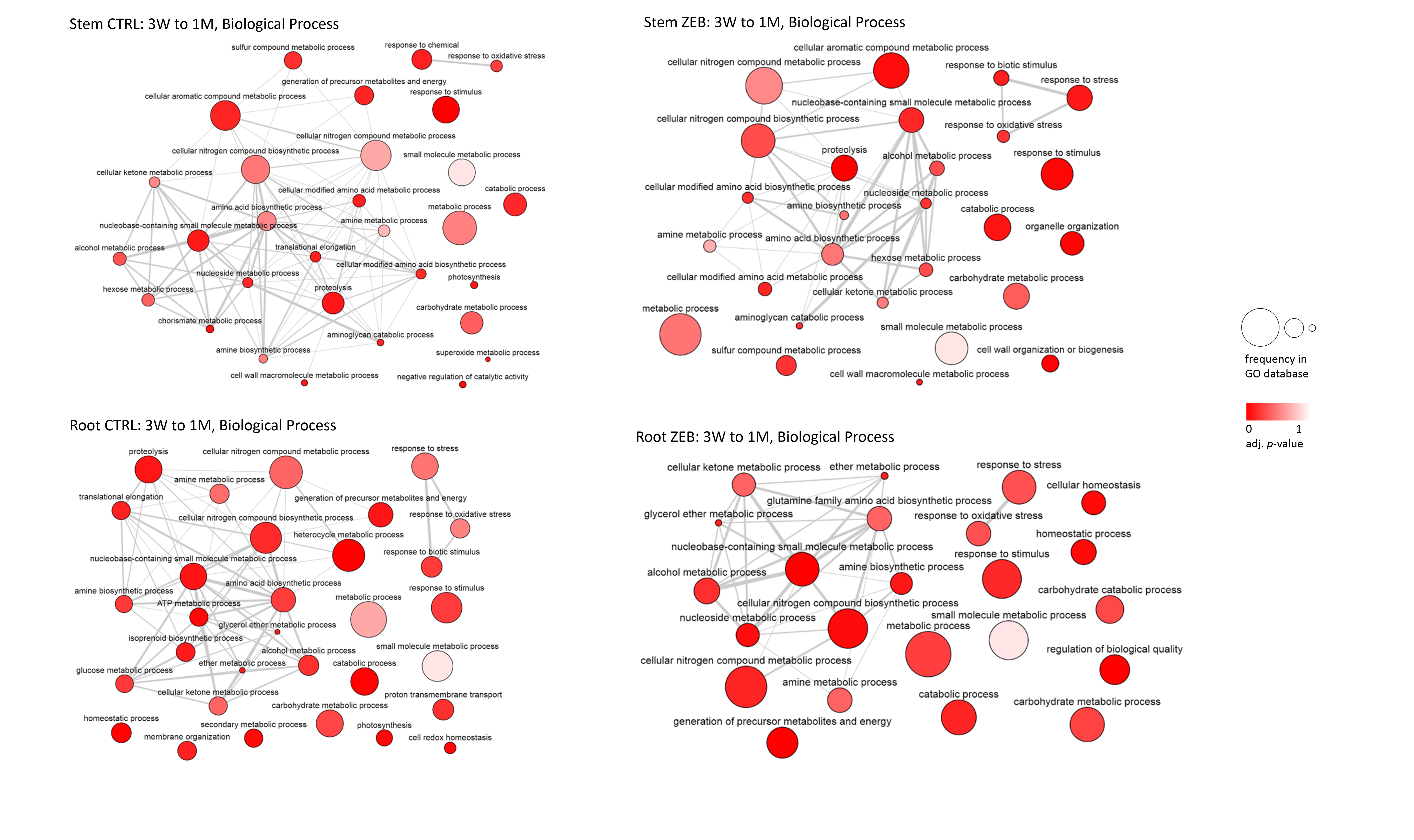

Supplement: Supplementary Figure 5 — Networks of the relations between the hierarchically highest GO terms (Biological Process) identified among the differentially accumulated proteins in zebularine-treated/untreated stems and roots in the transition from three weeks of recovery and one more month of growth in soil. Circle size indicates size of the term, red color indicates lower FDR. [file Image_5.png]

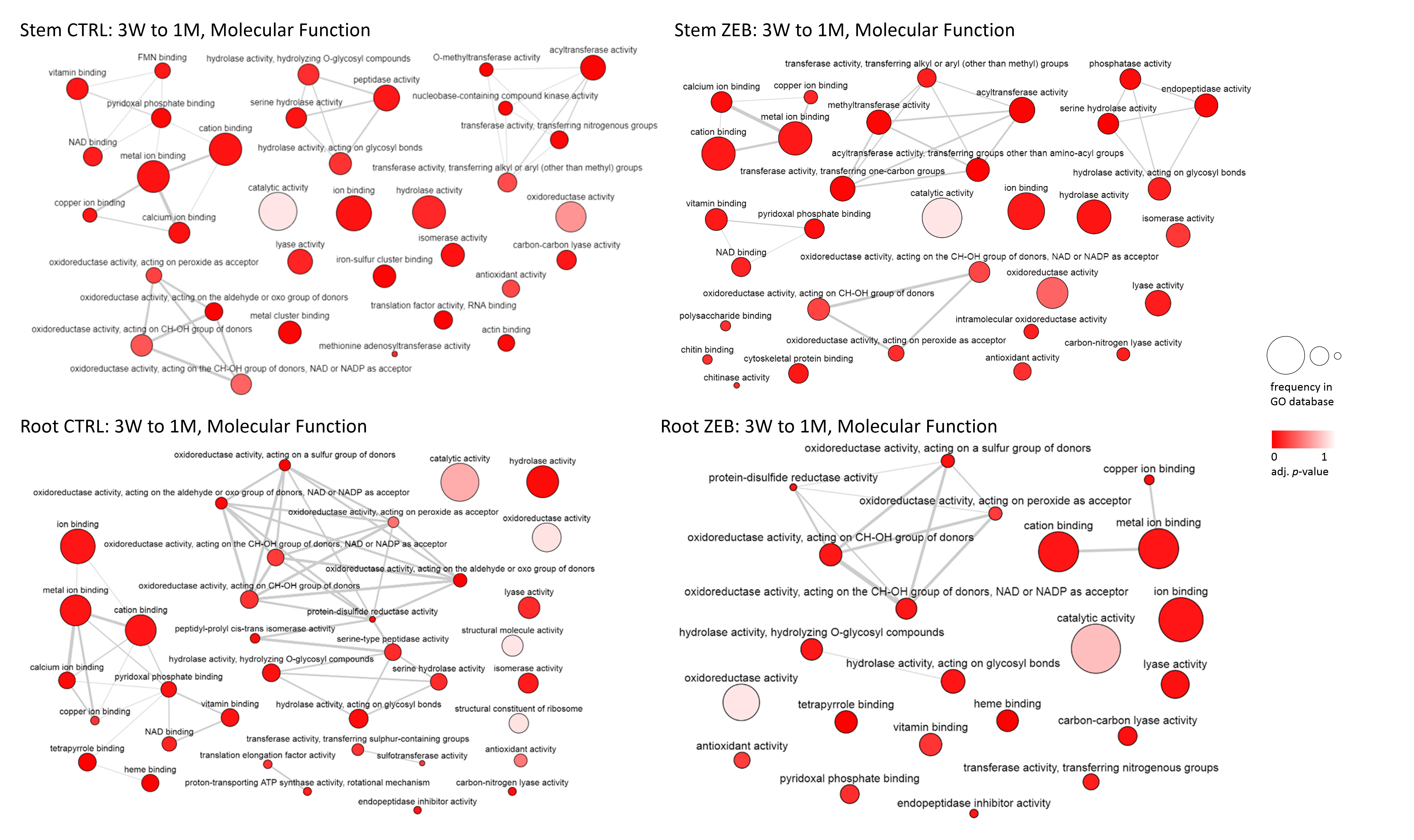

Supplement: Supplementary Figure 6 — Networks of the relations between the hierarchically highest GO terms (Molecular Function) identified among the differentially accumulated proteins in zebularine-treated/untreated stems and roots in the transition from three weeks of recovery and one more month of growth in soil. Circle size indicates size of the term, red color indicates lower FDR. [file Image_6.png]

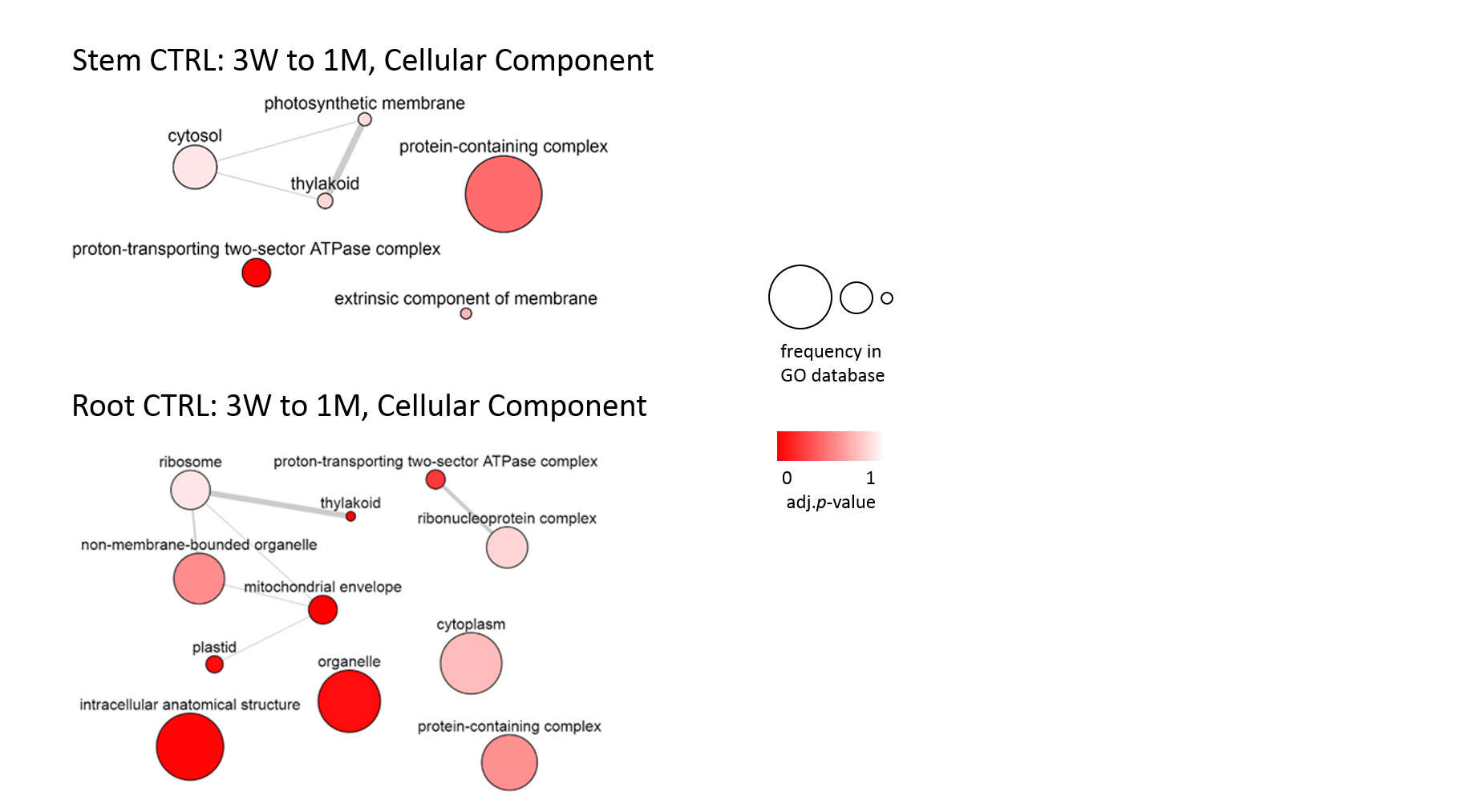

Supplement: Supplementary Figure 7 — Networks of the relations between the hierarchically highest GO terms (Cellular Component) identified among the differentially accumulated proteins in zebularine-treated/untreated stems and roots in the transition from three weeks of recovery and one more month of growth in soil. Circle size indicates size of the term, red color indicates lower FDR. [file Image_7.png]

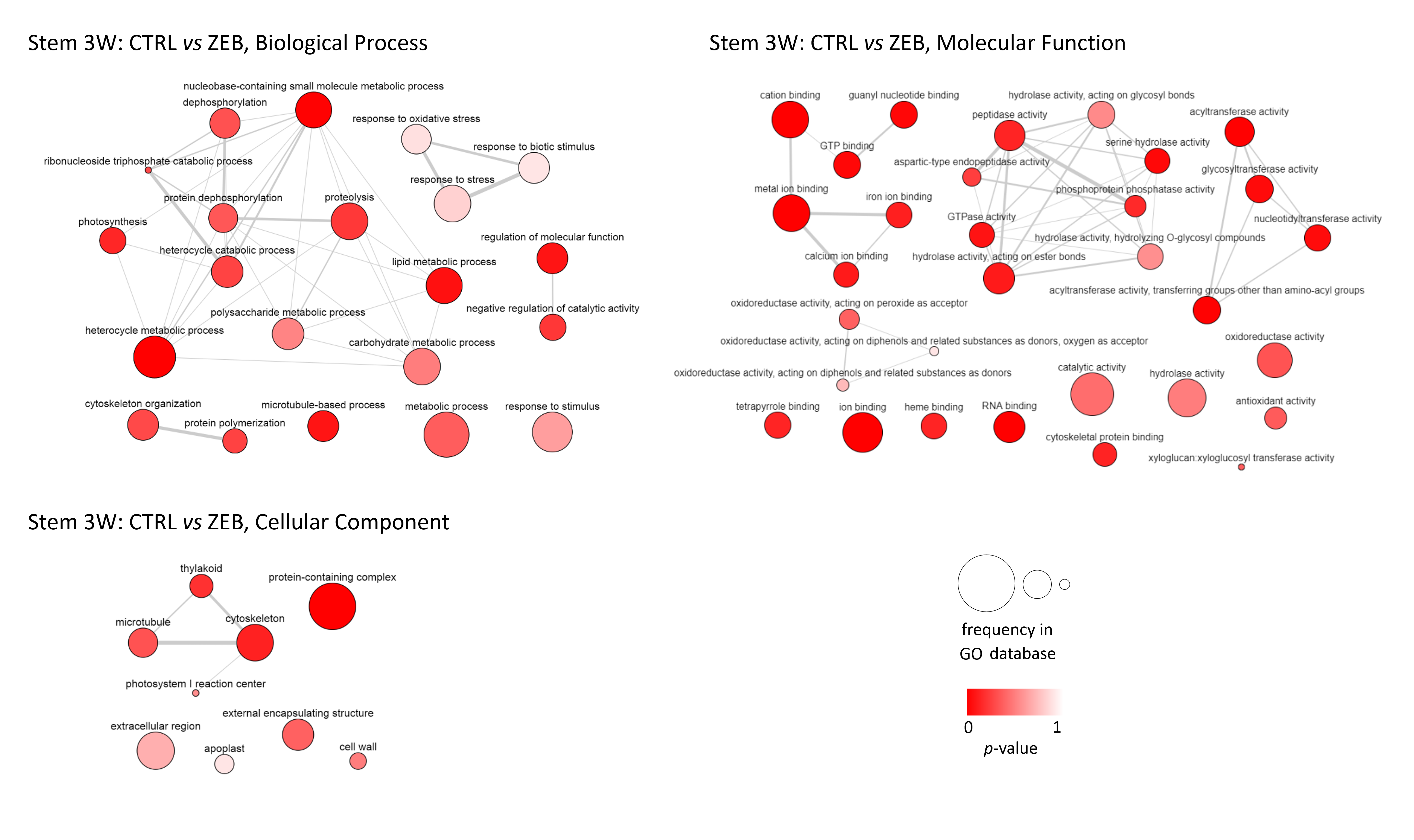

Supplement: Supplementary Figure 8 — Network of the relations between the hierarchically highest GO terms (Biological Process, Molecular Function, and Cellular Component) identified among the differentially accumulated proteins in stems after three weeks of recovery from zebularine. Circle size indicates size of the term, red color indicates lower p-value. [file Image_8.png]

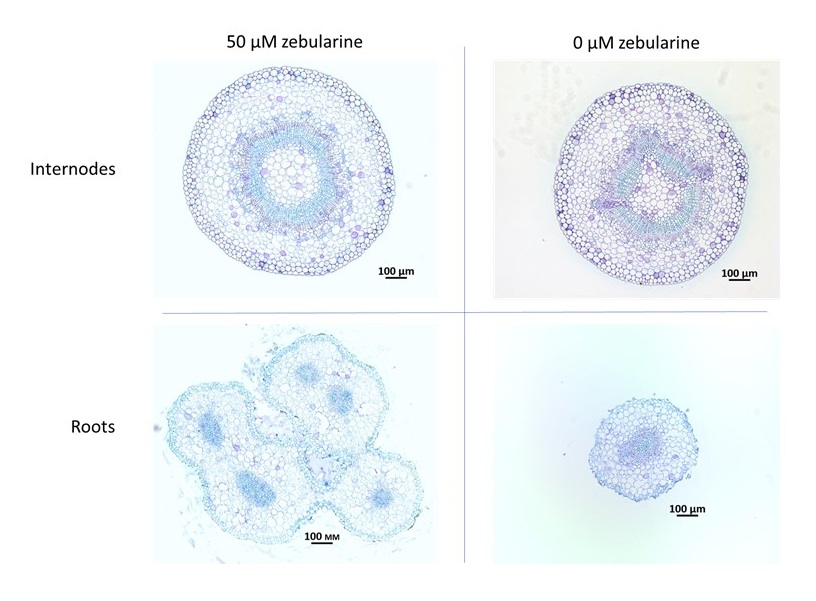

Supplement: Supplementary Figure 9 — Microscopy observations of roots and stems after three weeks of in vitro recovery, considering zebularine-treated and control plantlets. [file Image_9.jpeg]
